# Comparison of IT Neural Response Statistics with Simulations

**DOI:** 10.3389/fncom.2017.00060

**Published:** 2017-07-12

**Authors:** Qiulei Dong, Bo Liu, Zhanyi Hu

**Affiliations:** ^1^National Laboratory of Pattern Recognition, Institute of Automation, Chinese Academy of Sciences Beijing, China; ^2^Department of Artificial Intelligence, University of Chinese Academy of Sciences Beijing, China; ^3^Center for Excellence in Brain Science and Intelligence Technology, Chinese Academy of Sciences Beijing, China

**Keywords:** synthetic neuron response, single-neuron selectivity, population sparseness, response statistics

## Abstract

Lehky et al. (2011) provided a statistical analysis on the responses of the recorded 674 neurons to 806 image stimuli in anterior inferotemporalm (AIT) cortex of two monkeys. In terms of kurtosis and Pareto tail index, they observed that the population sparseness of both unnormalized and normalized responses is always larger than their single-neuron selectivity, hence concluded that the critical features for individual neurons in primate AIT cortex are not very complex, but there is an indefinitely large number of them. In this work, we explore an “inverse problem” by simulation, that is, by simulating each neuron indeed only responds to a very limited number of stimuli among a very large number of neurons and stimuli, to assess whether the population sparseness is always larger than the single-neuron selectivity. Our simulation results show that the population sparseness exceeds the single-neuron selectivity in most cases even if the number of neurons and stimuli are much larger than several hundreds, which confirms the observations in Lehky et al. (2011). In addition, we found that the variances of the computed kurtosis and Pareto tail index are quite large in some cases, which reveals some limitations of these two criteria when used for neuron response evaluation.

## 1. Introduction

In recent years, many researchers have investigated statistics of neuron responses in different visual cortical areas, since statistical characteristics of neurons are important to theories of object representation and population decoding (Riesenhuber and Poggio, 1999; Hinton et al., 2006; Lehky et al., 2007; Baldassi et al., 2013; Cadieu et al., 2014; Yamins et al., 2014; Dong et al., 2016; Chang and Tsao, 2017).

Single-neuron selectivity and population sparseness are two important characteristics of neuron responses, which have been extensively investigated in literatures (Lehky et al., 2005, 2011; Franco et al., 2007). Single-neuron selectivity is determined from single-neuron responses to all the stimuli, while population sparseness is determined from population responses to individual stimulus. In Lehky et al. (2005) and Tolhurst et al. (2009), single-neuron selectivity and population sparseness of neurons in the V1 area were investigated. Compared with low-level visual cortical areas, inferotemporal (IT) cortex, where complex stimulus features are required for the activation of IT neurons (Kobatake and Tanaka, 1994), is generally believed to be the final stage in object recognition (Gross, 2008). Franco et al. (2007) investigated the single-neuron selectivity and population sparseness of neurons in monkey IT cortex, and concluded that they were identical. In contrast to the results in Franco et al. (2007), Lehky et al. (2011) provided a statistical analysis on the responses of 674 monkey IT neurons to 806 stimulus images. They additionally performed simulation experiments on a synthetic set of responses generated by gamma distributions. And their results showed that single-neuron selectivity and population sparseness in monkey IT cortex were quite different. In their work, single-neuron selectivity and population sparseness were measured by kurtosis and Pareto tail index. They observed that the population sparseness is always larger than the single-neuron selectivity for both unnormalized and normalized responses, which is interpreted as that the critical features for individual neurons in primate anterior inferotemporalm (AIT) cortex are not very complex, but there are an indefinitely large number of such critical features. This is largely different from the traditional structural models of object recognition, where a small number of standard features are employed.

In this work, our goal is not to build quantitative predictive models of neuron responses, unlike Yamins et al. (2014) where a four-layered regression model is introduced to accurately predict monkey IT neuron responses, and Chang and Tsao (2017) where an axis model is proposed to decode face representation. Rather, our goal is to investigate the following “inverse problem” on the conclusions in Lehky et al. (2011) by simulation: Assuming each neuron only responds to a very limited number of stimuli among a large number of neurons and stimuli, whether the population sparseness is always larger than the single-neuron selectivity, especially when the number of neurons and stimuli increases. To our knowledge, there is no investigations on such an issue in the literature up to now. Considering it is impractical, even impossible, to record too many neuron responses, the above issues are addressed in this work with the synthetic neuron responses generated by two models separately, under varying neuron numbers, stimulus numbers, and noise levels.

With such synthetic neuron responses, our results support the interpretations in Lehky et al. (2011). In other words, if each neuron only selects a limited number of features and there are many different features, by both kurtosis and Pareto tail index, the population sparseness is larger than the single-neuron selectivity in most cases for both the two response generating models. Besides, we also observed that the variances of the computed kurtosis and Pareto tail index in some cases are quite large, which reveals some limitations of these two criteria when used for neuron response evaluation.

## 2. Methods

### 2.1. Overview

In this work, assuming the conclusions in Lehky et al. (2011) always hold true regardless of the number of neurons and stimuli, that is, the critical features for individual neurons in primate AIT cortex are not very complex, but there are an indefinitely large number of such features, we simulate a large number of neuron responses subject to this assumption under various conditions by varying the neuron number, the stimulus number, the noise level, and then use the same criteria, as did in Lehky et al. (2011) for monkey AIT neurons, to assess whether the population sparseness for the synthetic responses is always larger than the single-neuron selectivity by both the kurtosis criterion and the Pareto tail index criterion. Here, we only focus on the preservation of the relative magnitude order, not the computed absolute values, between the population sparseness and the single-neuron selectivity.

To make the simulation meaningful, the first crucial issue is how to simulate the neuron responses. Here, we adopt the following two neuron response generating models:

1. Assuming there are *N* stimuli and an upper-limit number of neuron responses *N*_max_ per neuron (*N*_max_ ≪ *N*), the exact response number for each neuron is generated at random from the set {1, 2, 3, …, *N*_max_} (explicitly controlling the small number of the critical features for each neuron), and the corresponding indices of the stimuli which activate a neuron, are determined at random from the set {1, 2, 3, …, *N*} (reflecting the fact that there are a large number of different critical features to which different neurons are tuned). Then, the response magnitude is generated at random under a predefined distribution, as described in Section 2.2.1. In addition, synthetic responses with neural correlation are generated by the Copula method (Hu et al., 2007).
2. As shown in Lehky et al. (2011), the IT single-neuron responses can be modeled by gamma distributions where large-magnitude responses have a high probability of occurring within a limited stimulus set, and noise can be modeled by Poisson noise. Hence, we also used the same model in Lehky et al. (2011) to generate synthetic responses with/without noise and neural correlation, as described in Section 2.2.2.

The advantage of the first model is that we can strictly control the maximum response number of individual neurons, which is set by the parameter *N*_max_. Its disadvantage is that the generated responses lack of any biological basis. As our problem is to investigate the “inverse problem,” we thought this lack of biological basis does not severely affect the validity of our work.

The advantage of the second model is that each neuron response does approximately satisfy the IT neuron responses in Lehky et al. (2011), but the maximum response number of individual neurons cannot be explicitly controlled. In the second model, another problem is that although single-neuron responses can be modeled by a gamma distribution, how to generate the two parameters (the shape and scale) of the gamma distribution for population neurons, is a difficult, even an open issue. In Lehky et al. (2011), these two parameters are separately generated also by two gamma distributions under fixed parameter settings. We found how to generate the two parameters of the gamma distribution of individual neurons could significantly affect the estimated kurtosis and Pareto tail index, which will be discussed in detail in Section 4.

Taking into account of the above points, we investigate both the two models in this work, and the final results by the two models are indeed similar in most cases, which further confirm the appropriateness of the interpretations in Lehky et al. (2011).

In the following subsections, we firstly describe the response generating methods derived from the two models in detail, and then introduce the related concepts, criteria, and the statistics of IT neuron responses in Lehky et al. (2011).

### 2.2. Methods for synthesizing neuron responses

At first, we give some notations to facilitate the subsequent descriptions:

**Notations:** Let $R\in R^{N\times M}$ represent a synthetic neuron response matrix without noise, where *N* is the number of the stimuli and *M* the number of the neurons. The *i*-th(*i* = 1, 2, …, *N*) row represents the responses of all the *M* neurons to the *i*-th stimulus, while the *j*-th(*j* = 1, 2, …, *M*) column represents the responses of the *j*-th neuron to all the *N* stimuli. Let $\tilde{R}\in R^{N\times M}$ represent the synthetic noisy response matrix of the noiseless response matrix *R*.

#### 2.2.1. Method-I for synthesizing neuron responses

Following the above mentioned first model, we assume that for each neuron, the number of the stimuli which can activate this neuron is no more than a preset positive integer *N*_max_, which is much smaller than the total number *N* of stimuli. That is to say, each neuron responds to no more than *N*_max_ stimuli with 0 < *N*_max_ ≪ *N*. And we assume that the number *M* of neurons is quite large. Then, the noiseless response matrix *R* is generated as follows:

S1 For the *j*-th(*j* = 1, 2, …, *M*) neuron, the number *N*_*j*_ of its selective stimuli is drawn from the discrete uniform distribution ϒ_*N*_max__ within the set {1, 2, 3, …, *N*_max_}.
S2 For the *j*-th(*j* = 1, 2, …, *M*) neuron, the indices $P_{j}^{k}\left(k\;=\;1,2,\ldots ,N_{j}\right)$ of the above mentioned *N*_*j*_ stimuli are selected randomly from the set {1, 2, 3, …, *N*}.
S3 For the *j*-th(*j* = 1, 2, …, *M*) neuron, its response $R_{P_{j}^{k},j}$ (*k* = 1, 2, …, *N*_*j*_) to the $P_{j}^{k}$-th stimulus is defined as $R_{P_{j}^{k},j}=\lambda_{j}exp\left(-\tau_{P_{j}^{k},j}\right)$, where the scalar λ_*j*_ is drawn from the continuous uniform distribution Ω_λ_ on the closed interval [1, λ_max_], λ_max_ is a preset threshold, and $\tau_{P_{j}^{k},j}$ is drawn from the continuous uniform distribution Ω_τ_ on the closed interval [0, 1].
S4 Constructing a noiseless response matrix *R*: Each of the synthetic responses $R_{P_{j}^{k},j}$ at Step S3 is assigned to the element at the $P_{j}^{k}$-th row and the *j*-th column of *R*, and the rest elements of *R* are set to 0.

Considering that noise is generally involved in the recorded neuron responses, we synthesize a noisy response matrix $\tilde{R}$ from the above noiseless matrix *R* via the following approach:

S1 Synthesizing a noise matrix δ: Its element δ_*i,j*_(*i* = 1, 2, …, *N, j* = 1, 2, …, *M*) is set as δ_*i,j*_ = max(0, ϕ_*i,j*_), where ϕ_*i,j*_ is drawn from the Gaussian distribution with mean μ and standard deviation σ. In the following parts, δ is called noise level.
S2 Constructing $\tilde{R}$: $\tilde{R}=R+\delta$.

#### 2.2.2. Method-II for synthesizing neuron responses

Here, we used the above mentioned second model (i.e., the simulation model in Lehky et al., 2011) to generate synthetic neuron responses with/without noise and neural correlation.

The original synthetic response matrix is constructed as: The synthetic responses of each neuron are generated by a gamma probability distribution. And for different neurons, their gamma distributions are different, whose parameters {*a, b*} are separately generated by two gamma distributions with fixed parameter settings [*a* = gamrnd(4.0, 0.5) and *b* = gamrnd(2.0, 0.5), where “gamrnd” is the Matlab gamma random number generator].

The synthetic noisy response matrix is constructed as: Each noisy response is generated by replacing its corresponding response in the above original response matrix by a Poisson-distributed random number whose mean value is the same to the original response.

The synthetic response matrix with neural correlation is constructed as: The correlation values between neurons are set to a constant, and then such synthetic response matrices are generated by the Copula method (Hu et al., 2007).

### 2.3. Concepts, criteria, and statistics of IT neuron responses

#### 2.3.1. Dataset in Lehky et al. (2011)

**Figure 1:**
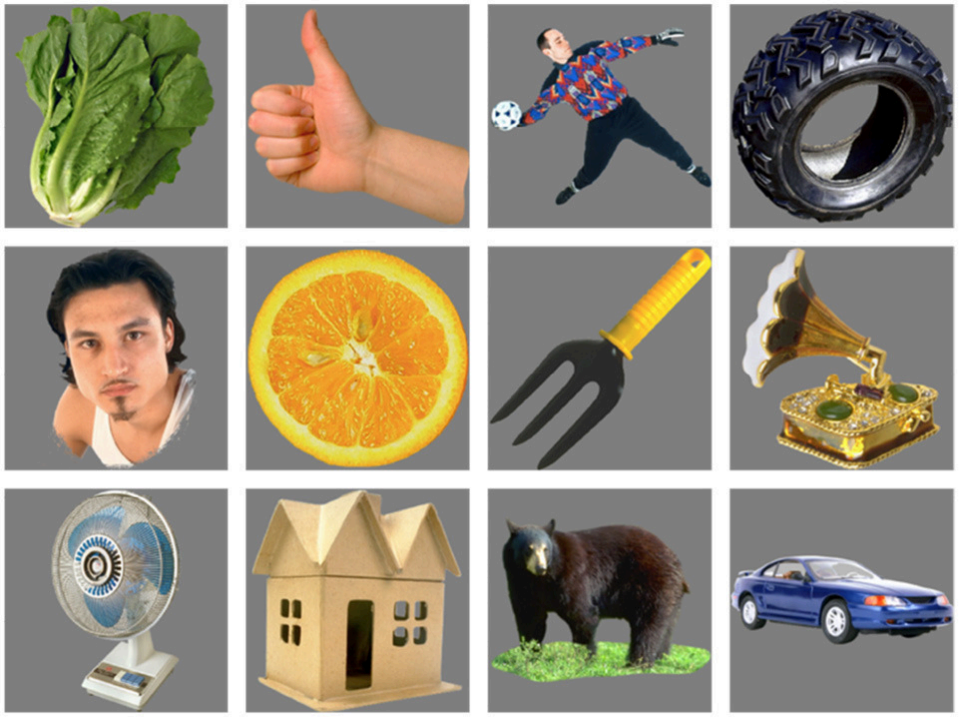
Example stimulus images from Lehky et al. (2011).

In Lehky et al. (2011), a 806 × 674 neuron response matrix is constructed, consisting of the recorded responses of 674 AIT neurons to 806 stimulus images of size 125 × 125. Each column of this response matrix is the responses of a single neuron to all the 806 images, and each row of this response matrix is the responses of all the 674 neurons to a single image. Some stimulus images are shown in Figure 1.

**Figure 2:**
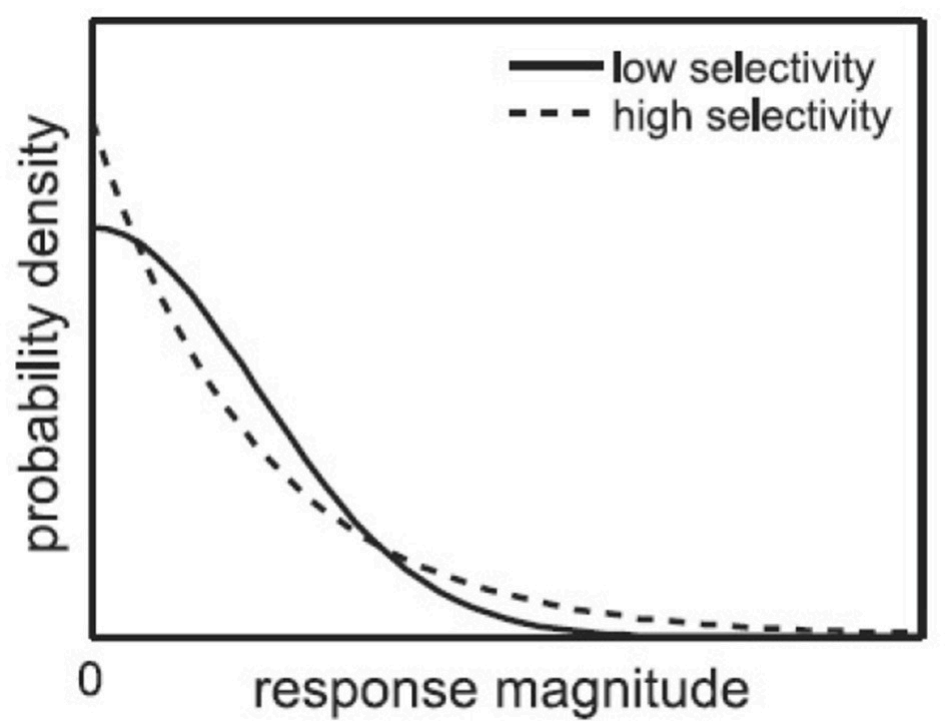
Probability density functions for high-selectivity and low-selectivity neuron responses (Lehky et al., 2011).

As introduced in Section 1, single-neuron selectivity and population sparseness are two related concepts of neuron responses. The single-neuron selectivity characterizes the distributions of single-neuron responses to all the stimuli, while the population sparseness characterizes the distributions of population responses to a single stimulus. For the above mentioned response matrix, each neuron has a fitted selectivity response probability density function, and each stimulus image has a population response probability density function. Figure 2 (from Lehky et al., 2011) shows an example of selectivity probability density functions, where the high-selectivity probability density function (dashed line) has a heavier upper tail than the low-selectivity probability density function (solid line). Given a probability density function for single-neuron responses (or population responses) as shown in Figure 2, if it has a substantial upper tail, it means high-selectivity responses have a larger probability of occurrence, in other words, the neuron is more selective (or the population response is more sparse).

In Lehky et al. (2011), the kurtosis and the Pareto tail index are used to measure the single-neuron selectivity and population sparseness of neurons, which are introduced, respectively, in the following subsections.

#### 2.3.2. Calculating single-neuron selectivity and population sparseness

##### 2.3.2.1. Kurtosis

Kurtosis (strictly speaking, excess kurtosis) is a measure of the “peakedness” of a probability distribution for both single-neuron selectivity and population sparseness in many existing works (Lehky et al., 2005, 2007, 2011; Tolhurst et al., 2009). It only depends on the shape of the distribution, and is independent of the mean or variance.

Kurtosis is defined as:

(1) $$\begin{array}{l} Kurt=\frac{\frac{1}{N}{\sum_{i=1}^{N}\left(r_{i}-\bar{r}\right)}^{4}}{{\left[\frac{1}{N}{\sum_{i=1}^{N}\left(r_{i}-\bar{r}\right)}^{2}\right]}^{2}}-3 \end{array}$$

where for single-neuron responses, *r*_*i*_ is the response of a neuron to the *i*-th image, *N* is the number of images; for population responses, *r*_*i*_ is the response of the *i*-th neuron to an image, *N* is the number of neurons. $\overset{®}{r}=\frac{1}{N}\overset{N}{\underset{i=1}{\sum }}r_{i}$ is the mean response.

**Normalization:** Neurons in a population may have different activation levels in some cases, then high population sparseness could arise as an artifact. To alleviate this problem, the normalized data $r_{i}^{n}$, which is obtained by dividing the response of each neuron by its mean response across all the stimulus images, is also used for calculating kurtosis on both single-neuron selectivity and population sparseness:

(2) $$\begin{array}{l} r_{i}^{n}=\frac{r_{i}}{\overset{®}{r}} \end{array}$$

where *r*_*i*_ is the response of a neuron to the *i*-th image, $\overset{®}{r}\;=\;\frac{1}{N}\overset{N}{\underset{i=1}{\sum }}r_{i}$ is the mean response across all *N* images. According to Equations (1) and (2), the normalization has no effect on single-neuron selectivity in principle, but does have an effect on population sparseness.

##### 2.3.2.2. Pareto tail index

The Pareto tail index (Pickands, 1975) is utilized to analyse large responses occurring on the upper tails of the probability density functions (PDFs). In Lehky et al. (2011), tail data were fitted with a generalized Pareto distribution by maximum likelihood.

The PDF for the generalized Pareto distribution is defined as:

(3) $$\begin{array}{l} p=f\left(r|k,\eta ,\theta \right)=\frac{1}{\theta }{\left(1+k\frac{r-\theta }{\eta }\right)}^{-1-\frac{1}{k}} \end{array}$$

where η is the scale, θ is the threshold where the upper tail of the probability density function starts, and *k* is a shape parameter quantifying heaviness of the tail, called the tail parameter.

Generally speaking, if the kurtosis is large, it means the density function most probably has a heavy tail. Similarly, if the Pareto tail index is large, the density function also has a heavy tail. Note that since kurtosis is a global measure of the shape of the entire probability distribution but not just the tail, measures of kurtosis and measures of Pareto tail index may be different in some cases. Note also that since population sparseness and single-neuron selectivity are evaluated by the same criteria and computed by the same formula, they are of no difference from the computational point of view.

**Remark:** Here, we would point out that for Method-II, since neuron responses are sampled from gamma distributions, whose shape and scale parameters are also sampled from two specific gamma distributions with the shape and scale parameters as described above, a large portion of such synthetic noiseless neuron responses are of a low magnitude, and accordingly, a large portion of the synthetic Poisson-noise responses by Method-II are zeros. Since for each neuron, the Pareto tail index is computed by the largest 10% of its responses as did in Lehky et al. (2011), we found the Pareto tail index is too sensitive to Poisson-noise responses, and the corresponding mean and median values cannot adequately describe the synthetic responses (these results are reported in Figure S4 of the Supplementary Materials). To remedy this problem caused by sampling at integer values in the Poisson distribution with parameter λ > 0, we used a truncated Gaussian distribution in place of the Poisson distribution to generate a noisy response $\tilde{r}$ in our experiments on Pareto tail index, i.e.,

(4) $$\begin{array}{l} \tilde{r}=\max\left(0,normrnd\left(\lambda ,\sqrt{\lambda }\right)\right) \end{array}$$

where “normrnd” is the Matlab Gaussian-distribution random number generator, and “$normrnd\left(\lambda ,\sqrt{\lambda }\right)$” generates random numbers subject to the Gaussian distribution with mean λ and standard deviation $\sqrt{\lambda }$. Here, the truncated Gaussian distribution is employed following the facts: (i) The mean and variance of $\tilde{r}$ generated from the normal distribution in Equation (4) are both equal to λ, same as those generated from the Poisson distribution with parameter λ; (ii) For sufficiently large values of λ, the normal distribution with mean λ and variance λ is an excellent approximation to the Poisson distribution with parameter λ (SOCR, 2017); (iii) The truncated Gaussian distribution $\tilde{r}=\max\left(0,normrnd\left(\lambda ,\sqrt{\lambda }\right)\right)$ characterizes a continuous variable, and it avoids possible “negative response” that could never occur in real neuron recordings. Note that although the probability of generating a positive sample using Equation (4) depends on λ, when λ ≥ 3, the probability of generating a “0” sample is lower than 4.2%. Since for the Pareto tail index estimation, we only compute the 10% of the largest responses, this truncated Gaussian will affect less on the Pareto tail index computation.

#### 2.3.3. General results in Lehky et al. (2011)

Lehky et al. (2011) showed that:

- For both the unnormalized and normalized neuron responses, the population sparseness is always greater than the single-neuron selectivity in terms of the mean kurtosis, median kurtosis, and mean Pareto tail index, as listed in Table 1 (these results are reported in Lehky et al., 2011).
- The above results are interpreted as that the critical features for individual neurons in primate AIT cortex are not quite complex, and there are an indefinitely large number of different critical features.

**Table 1:** Relative magnitude order of kurtosis and Pareto tail index between the single-neuron selectivity and the population sparseness on the recorded neuron responses in Lehky et al. (2011).

**Datasets**	**Kurtosis**				**Pareto tail index**			
	**Unnormalized data**		**Normalized data**		**Unnormalized data**		**Normalized data**	
	**Mean**	**Median**	**Mean**	**Median**	**Mean**	**Median**	**Mean**	**Median**
Single-neuron selectivity	3.50	1.88	3.50	1.88	−0.43	–	−0.43	–
Relative magnitude order	∧	∧	∧	∧	∧	–	∧	–
Population sparseness	12.51	9.61	17.35	7.98	−0.05	–	0.19	–

∧ *Indicates the selectivity is lower than the corresponding sparseness*.

## 3. Results

### 3.1. Parameter setting and data synthesis

In our experiments, all the codes are implemented in Matlab. We use the Matlab discrete uniform random number generator unidrnd, the Matlab random permutation function randperm, the Matlab continuous uniform random number generator unifrnd, the Matlab Gaussian random number generator normrnd, to generate synthetic responses in Method-I. And in Method-II, the Matlab gamma random number generator gamrnd is used both to generate synthetic responses and the two gamma-distribution parameters, and the Matlab Poisson random number generator poissrnd is used to generate noisy responses.

We synthesize the response matrices by Method-I and Method-II separately. For Method-I, in order to ensure that the number of neurons(or *M*) is sufficiently large as discussed in Section 2.2, *M* is set to 10,000, much larger than 674—the number of the recorded neurons in Lehky et al. (2011). And the number of the stimulus images (or *N*) is set to 2,000, much larger than 806—the number of the used stimuli in Lehky et al. (2011). In addition, in order to ensure that the critical features for individual neurons in primate AIT cortex are not very complex, the parameter *N*_max_ (the upper bound number of the stimuli activating a neuron) is set to {100, 200}, respectively, much smaller than the number *N* = 2,000 of the stimuli. Under the above parameter settings, the following response matrices are synthesized by Method-I:

- *R*_1_: a synthetic noiseless response matrix with *N*_max_ = 100 and λ_max_ = 50;
- *R*_2_: a synthetic noiseless response matrix with *N*_max_ = 200 and λ_max_ = 30;
- *R*_3_: a synthetic noiseless response matrix with *N*_max_ = 200 and λ_max_ = 50;
- ${\tilde{R}}_{3}^{1}$: a synthetic response matrix of low noise with mean μ = 0 and standard deviation σ = 1 from *R*_3_;
- ${\tilde{R}}_{3}^{2}$: a synthetic response matrix of medium noise with μ = 0 and σ = 3 from *R*_3_;
- ${\tilde{R}}_{3}^{3}$: a synthetic response matrix of high noise with μ = 15 and σ = 3 from *R*_3_.

And the following response matrices are synthesized by Method-II:

- *R*_4_: a 806 × 674 synthetic noiseless response matrix, which is of the same size as the response matrix used in Lehky et al. (2011). In this response matrix, the responses of each neuron are generated by a gamma distribution with its shape parameter *a* = gamrnd(4.0, 0.5) and scale parameter *b* = gamrnd(2.0, 0.5) as did in Lehky et al. (2011);
- ${\tilde{R}}_{4}^{1}$: a synthetic Poisson-noise response matrix from *R*_4_ as did in Lehky et al. (2011);
- ${\tilde{R}}_{4}^{2}$: a synthetic truncated-Gaussian-noise response matrix via Equation (4), where the mean and variance of the used Gaussian distribution are equal to the Poisson-distribution parameter used for generating ${\tilde{R}}_{4}^{1}$;
- *R*_5_: a 2,000 × 10,000 synthetic noiseless response matrix, where the responses of each neuron are generated by a gamma distribution with *a* = gamrnd(4.0, 0.5) and *b* = gamrnd(2.0, 0.5) as did in Lehky et al. (2011);
- ${\tilde{R}}_{5}^{1}$: a synthetic Poisson-noise response matrix from *R*_5_;
- ${\tilde{R}}_{5}^{2}$: a synthetic truncated-Gaussian-noise response matrix via Equation (4), where the mean and variance of the used Gaussian distribution are equal to the Poisson-distribution parameter used for generating ${\tilde{R}}_{5}^{1}$;

In addition, in order to evaluate the effect of neural correlation on the response statistics, we also generate the response matrices with correlation *r* by the Copula method (Hu et al., 2007). Considering that *r* = 0.2 could be the worst case of possible correlation of IT neuron responses as stated in Lehky et al. (2011), the following response matrices are generated with *r* = {0.1, 0.2}, respectively, as:

- *R*_5*r*1_: a 2,000 × 10,000 noiseless response matrix with *r* = 0.1 by Method-II;
- *R*_5*r*2_: a 2,000 × 10,000 noiseless response matrix with *r* = 0.2 by Method-II;
- ${\tilde{R}}_{5r1}^{1}$: a 2,000 × 10,000 Poisson-noise response matrix with *r* = 0.1 by Method-II;
- ${\tilde{R}}_{5r2}^{1}$: a 2,000 × 10,000 Poisson-noise response matrix with *r* = 0.2 by Method-II;
- ${\tilde{R}}_{5r1}^{2}$: a 2,000 × 10,000 truncated-Gaussian-noise response matrix with *r* = 0.1 by Method-II;
- ${\tilde{R}}_{5r2}^{2}$: 2,000 × 10,000 truncated-Gaussian-noise response matrix with *r* = 0.2 by Method-II;

### 3.2. Selectivity and sparseness by kurtosis

#### 3.2.1. Results on noiseless responses

The kurtosis values of the single-neuron responses in the synthetic noiseless response matrices *R*_1_, *R*_2_, *R*_3_, *R*_4_, and *R*_5_ (i.e., their column vectors) are computed for measuring the single-neuron selectivity. The kurtosis values of the synthetic population responses in *R*_1_, *R*_2_, *R*_3_, *R*_4_, and *R*_5_ (i.e., their row vectors) are computed for measuring the population sparseness. We also compute the selectivity and sparseness calculation on the normalized data via Equation (2).

**Figure 3:**
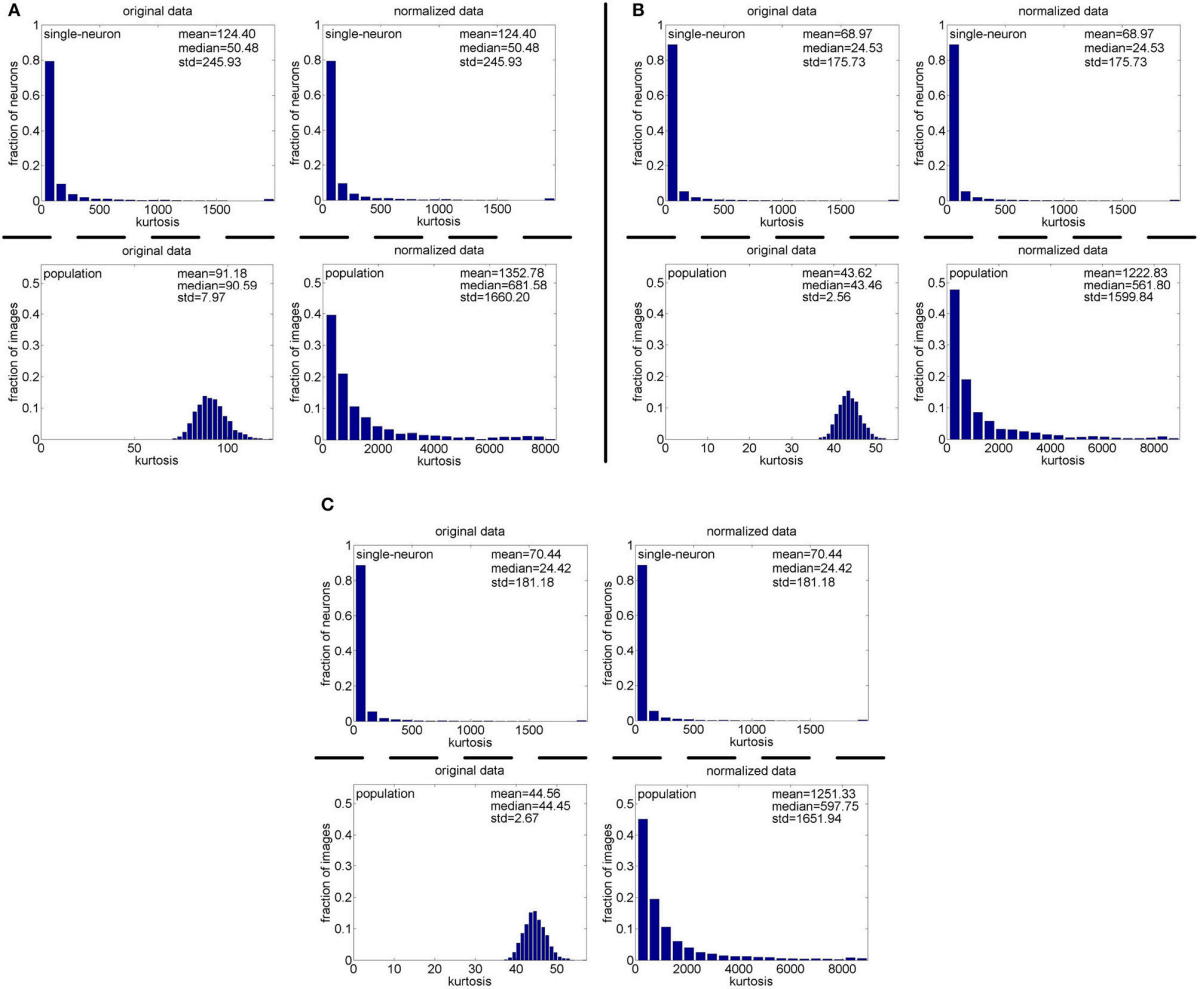
Single-neuron selectivity and population sparseness on the noiseless response matrices {*R*_1_, *R*_2_, *R*_3_} under Method-I: **(A)** Results on *R*_1_; **(B)** Results on *R*_2_; **(C)** Results on *R*_3_.

**Method-I:** The results on {*R*_1_, *R*_2_, *R*_3_} generated by Method-I are shown in Figure 3. Columns 2–7 of Table 2 list the relative magnitude order of kurtosis between the single-neuron selectivity and the population sparseness on the unnormalized and normalized synthetic responses from {*R*_1_, *R*_2_, *R*_3_}.

**Table 2:** Relative magnitude order of kurtosis between the single-neuron selectivity and the population sparseness on the unnormalized/normalized synthetic responses generated by Method-I.

	***R***_**1**_		***R***_**2**_		***R***_**3**_		${\tilde{R}}_{3}^{1}$		${\tilde{R}}_{3}^{2}$		${\tilde{R}}_{3}^{3}$	
	**Mean**	**Median**	**Mean**	**Median**	**Mean**	**Median**	**Mean**	**Median**	**Mean**	**Median**	**Mean**	**Median**
**UNNORMALIZED DATA**												
Single-neuron selectivity	124.40	50.48	68.97	24.53	70.44	24.42	37.91	19.76	18.19	13.23	10.40	9.03
Relative magnitude order	∨	∧	∨	∧	∨	∧	∧	∧	∧	∧	∧	∧
Population sparseness	91.18	90.59	43.62	43.46	44.56	44.45	43.01	42.92	33.25	33.20	21.27	21.26
**NORMALIZED DATA**												
Single-neuron selectivity	124.40	50.48	68.97	24.53	70.44	24.42	37.91	19.76	18.19	13.23	10.40	9.03
Relative magnitude order	∧	∧	∧	∧	∧	∧	∧	∧	∧	∧	∧	∧
Population sparseness	1,352.78	681.58	1,222.83	561.80	1,251.33	597.75	65.02	60.30	26.78	26.27	18.17	18.18

∨ Indicates the single-neuron selectivity is larger than the corresponding population sparseness, while

∧ *Indicates the single-neuron selectivity is smaller than the corresponding population sparseness*.

As seen from Figure 3, the computed mean and median kurtosis values of the unnormalized single-neuron responses are the same as those of the normalized single-neuron responses, because normalization does not affect the single-neuron selectivity as discussed in Section 2.3. Due to the computational nature of kurtosis, neurons responding to a smaller number of stimuli (much smaller than *N*_max_) would usually have a bigger kurtosis. And among the 10,000 single-neuron response vectors generated by Method-I, we observed that there usually exist a few neurons which only respond to a very small number of stimuli (much smaller than *N*_max_) and whose kurtosis values for single-neuron selectivity are dozens of times larger than the mean and median kurtosis values, hence resulting in a large standard deviation (denoted as “std” in Figure 3) of kurtosis of the single-neuron responses. In addition, the computed mean and median kurtosis values of the unnormalized population responses are much smaller than those of the normalized population responses, mainly because a few synthetic neurons in {*R*_1_, *R*_2_, *R*_3_} are activated by quite a small number of the stimuli, i.e., their responses to most of the stimuli are 0. This means that the mean response for each of these neurons is very small, and a few responses of these neurons are unusually amplified after normalization as discussed in Section 2.3, resulting in a large kurtosis of population sparseness as well as a large standard deviation of kurtosis of the normalized population responses.

In addition, on the three noiseless response matrices, the mean kurtosis of the population sparseness is smaller than that of the single-neuron selectivity for the unnormalized data, but the median kurtosis of the population sparseness is larger than that of the single-neuron selectivity for the unnormalized data. However, both the mean and median kurtosis values of the population sparseness are larger than those of the single-neuron selectivity for the normalized data.

**Figure 4:**
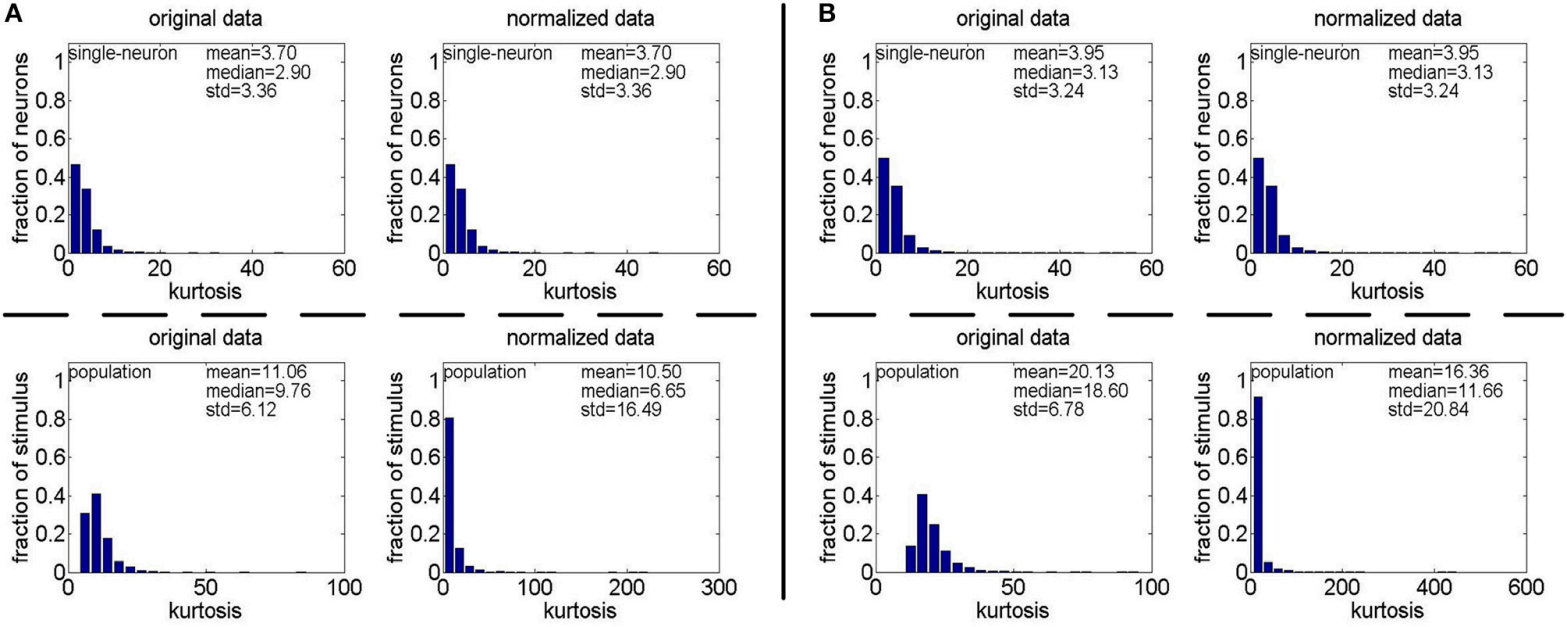
Single-neuron selectivity and population sparseness on the noiseless response matrices *R*_4_ and *R*_5_ under Method-II: **(A)** Results on *R*_4_; **(B)** Results on *R*_5_.

**Method-II:** The results on {*R*_4_, *R*_5_} generated by Method-II (the response generating model used in Lehky et al., 2011) are shown in Figure 4. As is seen, the computed standard deviations of kurtosis for both single-neuron selectivity and population sparseness are relatively large. In addition, Columns 2–5 of Table 3 list the relative magnitude order of kurtosis between the single-neuron selectivity and the population sparseness on the unnormalized and normalized synthetic responses from {*R*_4_, *R*_5_}. As is seen, both the mean and median kurtosis values of the population sparseness are larger than those of the single-neuron selectivity for the unnormalized and normalized data. That is to say, although the the absolute magnitudes of the computed mean and median kurtosis for the single-neuron selectivity and the population sparseness on {*R*_4_, *R*_5_} are different from those on {*R*_1_, *R*_2_, *R*_3_} generated by Method-I, the relative magnitude order of kurtosis on {*R*_4_, *R*_5_} is consistent with that on {*R*_1_, *R*_2_, *R*_3_} in most cases.

**Table 3:** Relative magnitude order of kurtosis between the single-neuron selectivity and the population sparseness on the unnormalized/normalized synthetic responses generated by Method-II.

	***R***_**4**_		***R***_**5**_		${\tilde{R}}_{4}^{1}$		${\tilde{R}}_{5}^{1}$		${\tilde{R}}_{4}^{2}$		${\tilde{R}}_{5}^{2}$	
	**Mean**	**Median**	**Mean**	**Median**	**Mean**	**Median**	**Mean**	**Median**	**Mean**	**Median**	**Mean**	**Median**
**UNNORMALIZED DATA**												
Single-neuron selectivity	3.70	2.90	3.95	3.13	4.67	3.30	4.90	3.73	4.34	3.29	4.28	3.39
Relative magnitude order	∧	∧	∧	∧	∧	∧	∧	∧	∧	∧	∧	∧
Population sparseness	11.06	9.76	20.13	18.60	13.43	11.08	15.63	14.74	16.24	12.90	15.38	14.57
**NORMALIZED DATA**												
Single-neuron selectivity	3.70	2.90	3.95	3.13	4.67	3.30	4.90	3.73	4.34	3.29	4.28	3.39
Relative magnitude order	∧	∧	∧	∧	∧	∧	∧	∧	∧	∧	∧	∧
Population sparseness	10.50	6.65	16.36	11.66	28.04	12.01	69.81	37.05	9.91	6.80	12.24	9.20

∧ *Indicates the selectivity is lower than the corresponding sparseness*.

#### 3.2.2. Results on responses with noise

The kurtosis values of the unnormalized and normalized data in the synthetic noisy response matrices $\left\{{\tilde{R}}_{3}^{1},{\tilde{R}}_{3}^{2},{\tilde{R}}_{3}^{3},{\tilde{R}}_{4}^{1},{\tilde{R}}_{4}^{2},{\tilde{R}}_{5}^{1},{\tilde{R}}_{5}^{2}\right\}$ are computed for measuring the population sparseness and the single-neuron selectivity.

**Figure 5:**
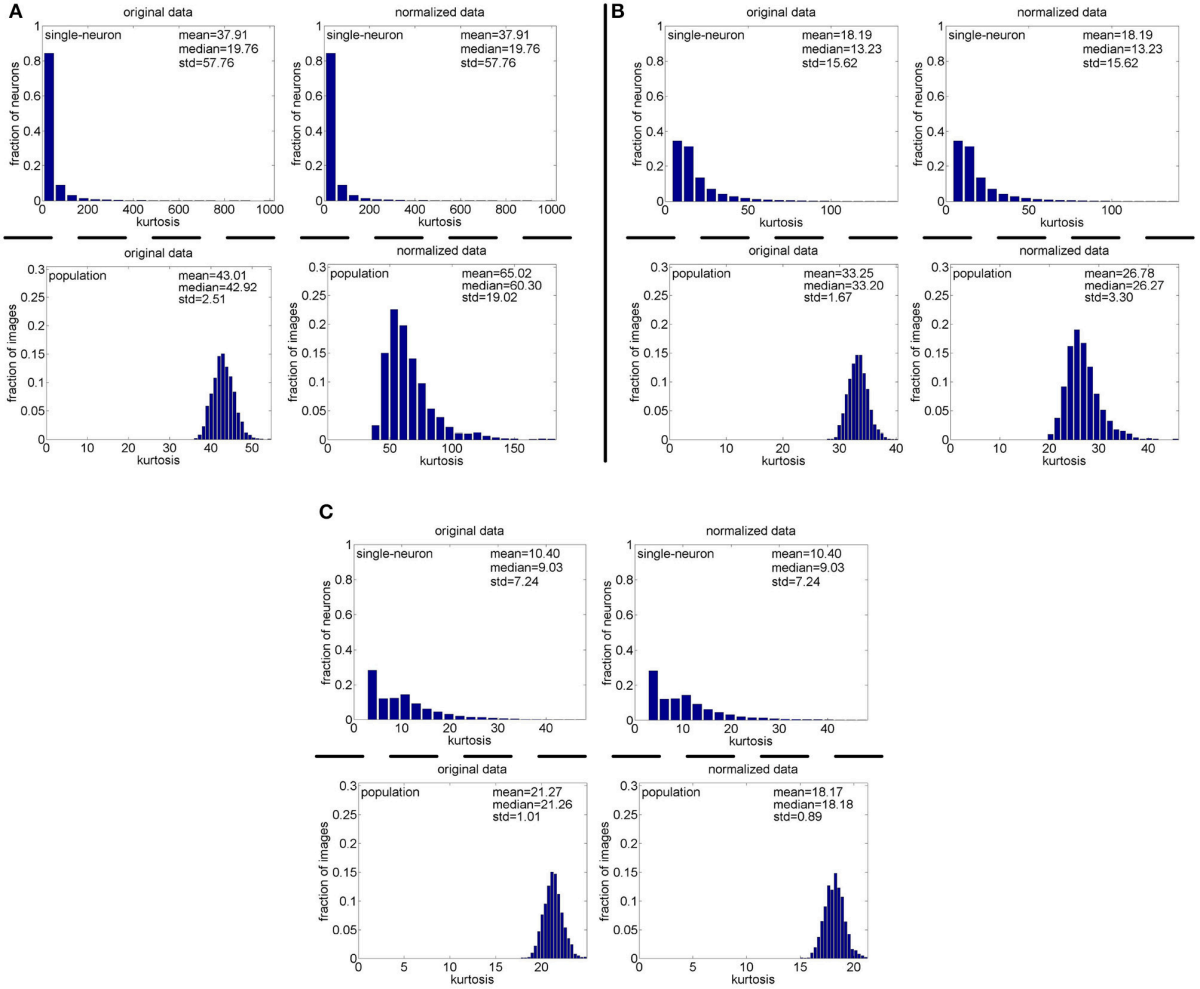
Single-neuron selectivity and population sparseness on the noisy response matrices $\left\{{\tilde{R}}_{3}^{1},{\tilde{R}}_{3}^{2},{\tilde{R}}_{3}^{3}\right\}$ under Method-I: **(A)** Results on ${\tilde{R}}_{3}^{1}$; **(B)** Results on ${\tilde{R}}_{3}^{2}$; **(C)** Results on ${\tilde{R}}_{3}^{3}$.

**Method-I:** The results on $\left\{{\tilde{R}}_{3}^{1},{\tilde{R}}_{3}^{2},{\tilde{R}}_{3}^{3}\right\}$ generated by Method-I are shown in Figure 5 and Columns 8–13 of Table 2. As seen from Figure 5A, the computed mean and median kurtosis values for the unnormalized population responses are smaller than those for the normalized population responses in ${\tilde{R}}_{3}^{1}$ with low noise. However, as seen from Figures 5B,C, with the increase of noise level, the kurtosis of the unnormalized population responses becomes larger than that of the normalized population responses in both ${\tilde{R}}_{3}^{2}$ with medium noise and ${\tilde{R}}_{3}^{3}$ with high noise. This is mainly because the mean value of each synthetic single-neuron response becomes relatively large with the increase of noise, and the normalization with a larger mean value could transform the original neuron responses with different levels of activation into the responses under an approximately single level of activation.

As seen from Table 2, for both the unnormalized and normalized data from the three noisy response matrices, the mean and median kurtosis values of the population responses are both larger than those of the single-neuron responses.

**Figure 6:**
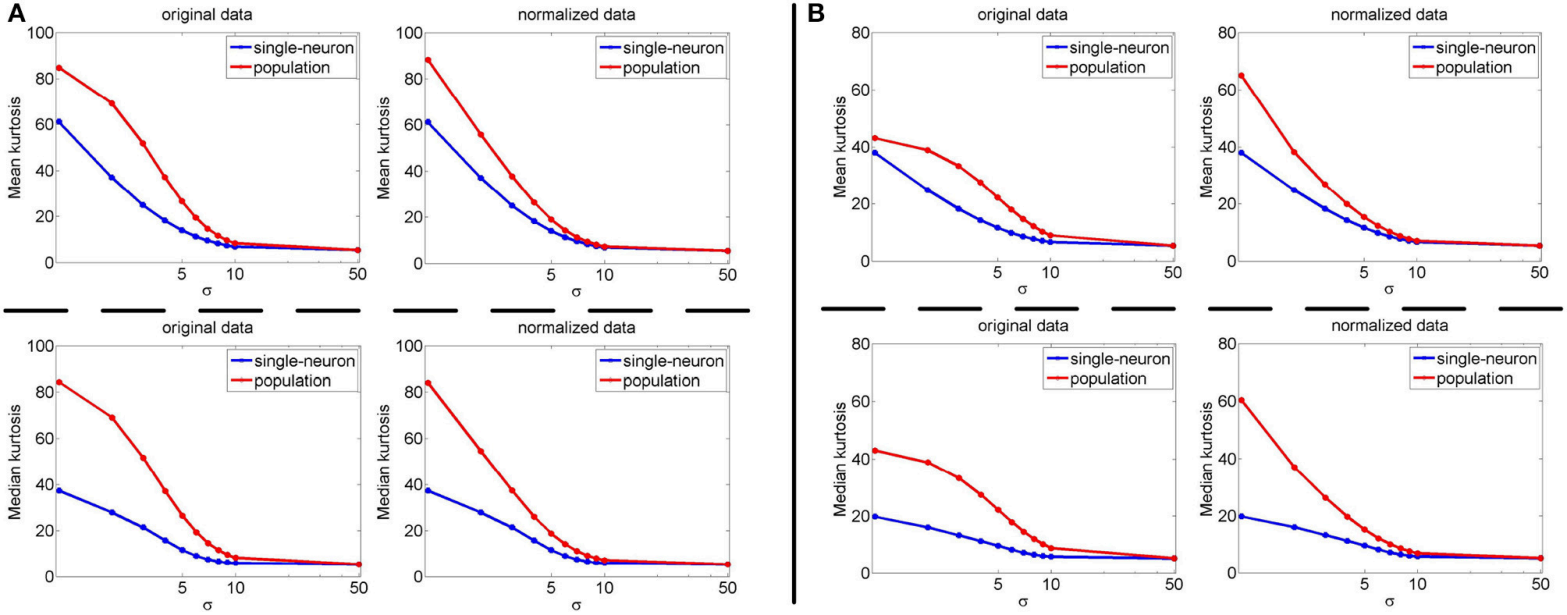
Mean and median kurtosis for unnormalized/normalized single-neuron responses and population responses with different levels of noise (μ = 0 and σ = {1, 2, 3, 4, 5, 6, 7, 8, 9, 10, 50}) under Method-I: **(A)** Results by corrupting *R*_1_; **(B)** Results by corrupting *R*_3_.

In addition, we also test the synthetic responses by separately corrupting *R*_1_(*N*_max_ = 100) and *R*_3_(*N*_max_ = 200) with different levels of noise (μ = 0 and σ = {1, 2, 3, 4, 5, 6, 7, 8, 9, 10, 50}), and the corresponding results are shown in Figure 6 with its x-axis plotted on a logarithmic scale. As seen from Figure 6, under different levels of noise and different upper-limit numbers (*N*_max_) of neuron responses, the calculated population sparseness is always larger than the single-neuron selectivity for both the unnormalized and normalized data. In addition, with the increase of the noise level, the calculated mean and median kurtosis decreases accordingly. When the noise level is too high, both the mean and median kurtosis values become saturated, because more and more real responses in *R*_1_ and *R*_3_ are close to or even lower than the noise under such conditions.

**Figure 7:**
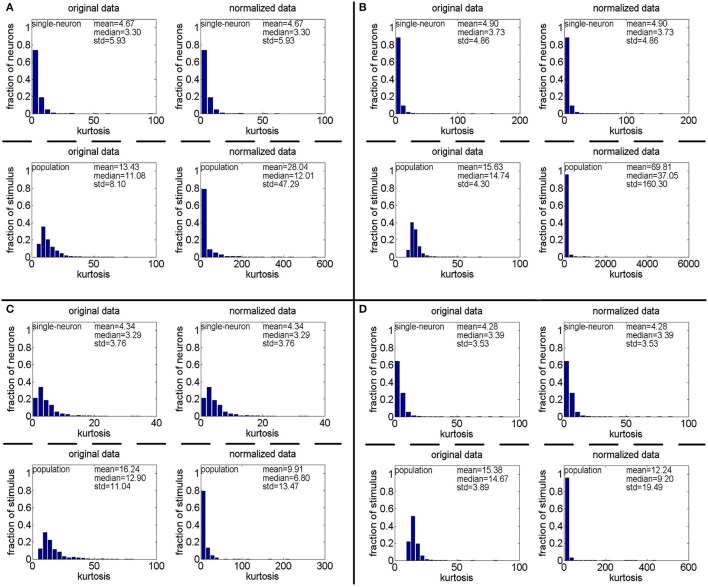
Single-neuron selectivity and population sparseness on the noisy matrices $\left\{{\tilde{R}}_{4}^{1},{\tilde{R}}_{5}^{1},{\tilde{R}}_{4}^{2},{\tilde{R}}_{5}^{2}\right\}$ under Method-II: **(A)** Results on ${\tilde{R}}_{4}^{1}$; **(B)** Results on ${\tilde{R}}_{5}^{1}$; **(C)** Results on ${\tilde{R}}_{4}^{2}$; **(D)** Results on ${\tilde{R}}_{5}^{2}$.

**Method-II:** The results on $\left\{{\tilde{R}}_{4}^{1},{\tilde{R}}_{4}^{2},{\tilde{R}}_{5}^{1},{\tilde{R}}_{5}^{2}\right\}$ generated by Method-II are shown in Figure 7, and the corresponding relative magnitudes orders of kurtosis are reported in Columns 8–13 of Table 3. As is seen, for both the unnormalized and normalized data, the computed mean and median kurtosis values for the population sparseness are always larger than those for the single-neuron selectivity. These results are consistent with the above results on the responses generated by Method-I.

#### 3.2.3. Results on responses with neural correlation

The kurtosis values of the unnormalized and normalized responses from $\left\{R_{5r1},R_{5r2},{\tilde{R}}_{5r1}^{1},{\tilde{R}}_{5r2}^{1},{\tilde{R}}_{5r1}^{2},{\tilde{R}}_{5r2}^{2}\right\}$ are computed for measuring the population sparseness and the single-neuron selectivity under neural correlation. The corresponding results are reported in Table 4 (also in Figure S1 of the Supplementary Materials). As is seen, for both the unnormalized and normalized data, the computed mean and median kurtosis values for the population sparseness are larger than those for the single-neuron selectivity. These results are consistent with the reported results on the responses without neural correlation in Table 3. In sum, under the correlation *r* = 0.2, the correlation does not change the relative magnitude order of kurtosis.

**Table 4:** Relative magnitude order of kurtosis between the single-neuron selectivity and the population sparseness on the unnormalized/normalized synthetic responses with neural correlation.

	***R***_**5***r***1**_		***R***_**5***r***2**_		${\tilde{R}}_{5r1}^{1}$		${\tilde{R}}_{5r2}^{1}$		${\tilde{R}}_{5r1}^{2}$		${\tilde{R}}_{5r2}^{2}$	
	**Mean**	**Median**	**Mean**	**Median**	**Mean**	**Median**	**Mean**	**Median**	**Mean**	**Median**	**Mean**	**Median**
**UNNORMALIZED DATA**												
Single-neuron selectivity	3.91	3.16	3.93	3.13	4.86	3.65	4.90	3.65	4.32	3.40	4.38	3.43
Relative magnitude order	∧	∧	∧	∧	∧	∧	∧	∧	∧	∧	∧	∧
Population sparseness	19.02	17.92	16.34	15.32	18.81	16.75	14.42	13.44	14.20	13.46	14.11	13.36
**NORMALIZED DATA**												
Single-neuron selectivity	3.91	3.16	3.93	3.13	4.86	3.65	4.90	3.65	4.32	3.40	4.38	3.43
Relative magnitude order	∧	∧	∧	∧	∧	∧	∧	∧	∧	∧	∧	∧
Population sparseness	12.00	9.41	11.96	8.55	102.15	44.07	108.55	39.78	13.02	8.81	10.46	7.92

∧ *Indicates the selectivity is lower than the corresponding sparseness*.

#### 3.2.4. Results under different neuron numbers and stimulus numbers

Since the relative magnitude order of kurtosis of the population sparseness with respect to the single-neuron selectivity on the synthetic responses generated by Method-I is in agreement with that by Method-II in most cases as demonstrated by the above results, in this subsection, we only calculate the single-neuron selectivity and the population sparseness under varying dataset sizes resampled from the synthetic response matrices $\left\{R_{3},{\tilde{R}}_{3}^{1},{\tilde{R}}_{3}^{2},{\tilde{R}}_{3}^{3}\right\}$ generated by Method-I (some results on the synthetic response matrices by Method-II are reported in Figures S2, S3 of the Supplementary Materials). Note that as done in Lehky et al. (2011), when dealing with the single-neuron responses, dataset size refers to the number of the stimulus images tested on each neuron, and when dealing with the population responses, dataset size refers to the number of the neurons in the population.

**Figure 8:**
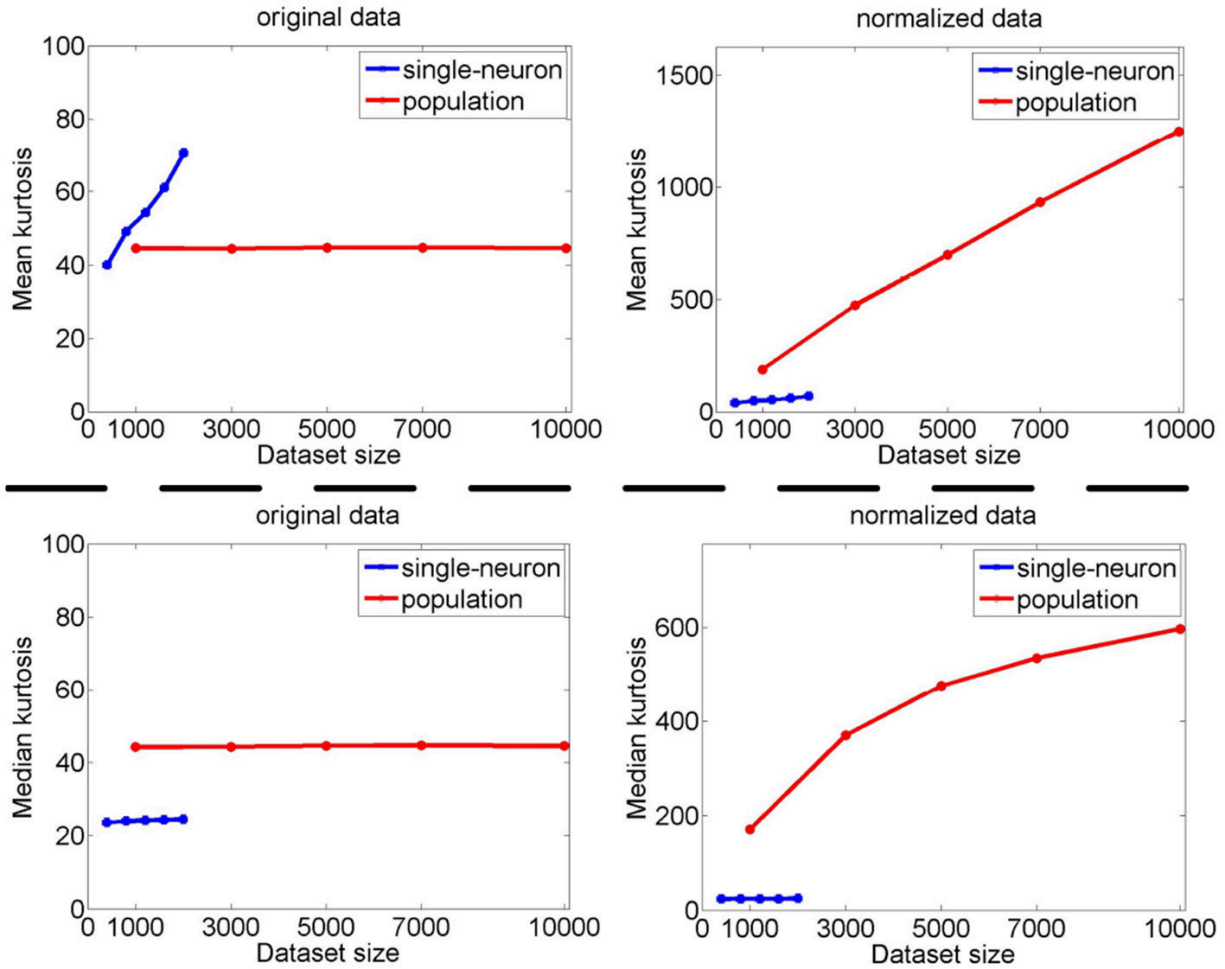
Mean and median kurtosis for single-neuron responses and population responses with different numbers of stimuli and neurons on *R*_3_ under Method-I.

**Figure 9:**
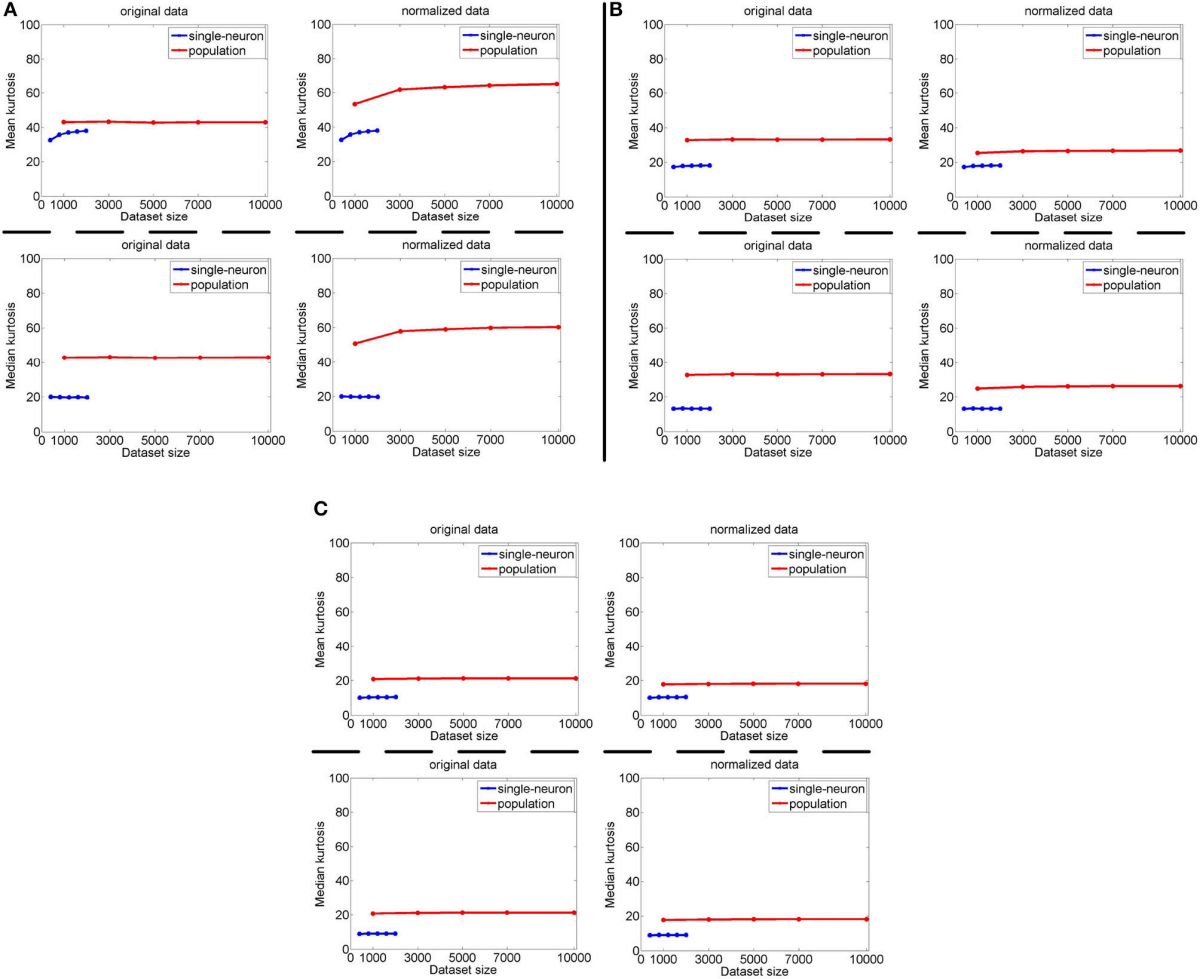
Mean and median kurtosis for single-neuron responses and population responses with different numbers of stimuli and neurons on $\left\{{\tilde{R}}_{3}^{1},{\tilde{R}}_{3}^{2},{\tilde{R}}_{3}^{3}\right\}$ under Method-I: **(A)** Results on ${\tilde{R}}_{3}^{1}$; **(B)** Results on ${\tilde{R}}_{3}^{2}$; **(C)** Results on ${\tilde{R}}_{3}^{3}$.

The image subset sizes of [400, 800, 1, 200, 1, 600, 2, 000] are tested for single-neuron responses, and the neuron subset sizes of [1, 000, 3, 000, 5, 000, 7, 000, 10, 000] are tested for population responses. Under each image-size and neuron-size combination, the sampling is independently done 10 times, and the mean value of the 10 estimated kurtosis values is used as the final kurtosis. Figures 8, 9 show the mean and median kurtosis values of the unnormalized and normalized responses under different numbers of stimuli and neurons on the noiseless response matrix *R*_3_ and the noisy matrices $\left\{{\tilde{R}}_{3}^{1},{\tilde{R}}_{3}^{2},{\tilde{R}}_{3}^{3}\right\}$, respectively.

From Figure 8, for the noiseless response matrix *R*_3_ under different neuron-stimulus combinations, the mean kurtosis of the unnormalized population responses is smaller than that of the unnormalized single-neuron responses, but the median kurtosis of the unnormalized population responses is larger than that of the unnormalized single-neuron responses. Both the mean and median kurtosis values of the normalized population responses in *R*_3_ are always larger than those of the normalized single-neuron responses. These results are consistent with the corresponding kurtosis results in Section 3.2.1.

For both the normalized and unnormalized data from the noisy response matrices $\left\{{\tilde{R}}_{3}^{1},{\tilde{R}}_{3}^{2},{\tilde{R}}_{3}^{3}\right\}$ under different neuron-stimulus combinations, the mean and median kurtosis values of the population responses are always larger than those of the single-neuron responses, which are consistent with the corresponding kurtosis results in Section 3.2.2.

It is noted that the mean and median kurtosis values of the normalized population responses from the noiseless response matrix *R*_3_ are much larger than those from the noisy matrices $\left\{{\tilde{R}}_{3}^{1},{\tilde{R}}_{3}^{2},{\tilde{R}}_{3}^{3}\right\}$. This is mainly because: (i) According to the approach for generating *R*_3_ in Section 2.2.1, a few of the synthetic neurons are activated by quite a small number of stimuli, i.e., their responses to most of the stimuli are 0. This means that the mean responses of these neurons across all the stimuli are tiny so that a few responses of these neurons to the stimuli are unusually amplified after normalization, resulting in large kurtosis values of population sparseness; (ii) The mean value of each synthetic single-neuron response in $\left\{{\tilde{R}}_{3}^{1},{\tilde{R}}_{3}^{2},{\tilde{R}}_{3}^{3}\right\}$ becomes relatively large due to the added noise, and normalization with such a larger mean value could transform the original neuron responses with different levels of activation into responses under an approximately single level of activation, resulting in moderate kurtosis values of population sparseness.

The above computed mean kurtosis (median kurtosis) also indicates that the relative magnitude order of kurtosis of the population sparseness with respect to the single-neuron selectivity does not change under different numbers of stimuli and neurons. In addition, except for the noiseless case, the estimated absolute values only change mildly under different numbers of stimuli and neurons and under a given noise level, although the estimated values under different noise levels do change significantly. These results imply that the reported results under 804 stimuli and 674 neurons in Lehky et al. (2011) have revealed the essential features of larger AIT neuron populations to some extent. In other words, if more AIT neurons were recorded, the computed relative magnitude order of kurtosis between the single-neuron selectivity and the population sparseness in Lehky et al. (2011) could not change much.

### 3.3. Pareto tail index

Pareto tail index (PTI) is another measure of single-neuron selectivity and population sparseness. In this subsection, Generalized Pareto distributions (GPDs) are fitted for both the probability distribution function of single-neuron responses and the probability distribution function of the population responses. The PTI-values of the single-neuron responses and population responses are computed, respectively.

#### 3.3.1. Pareto tail index for noiseless responses

**Figure 10:**
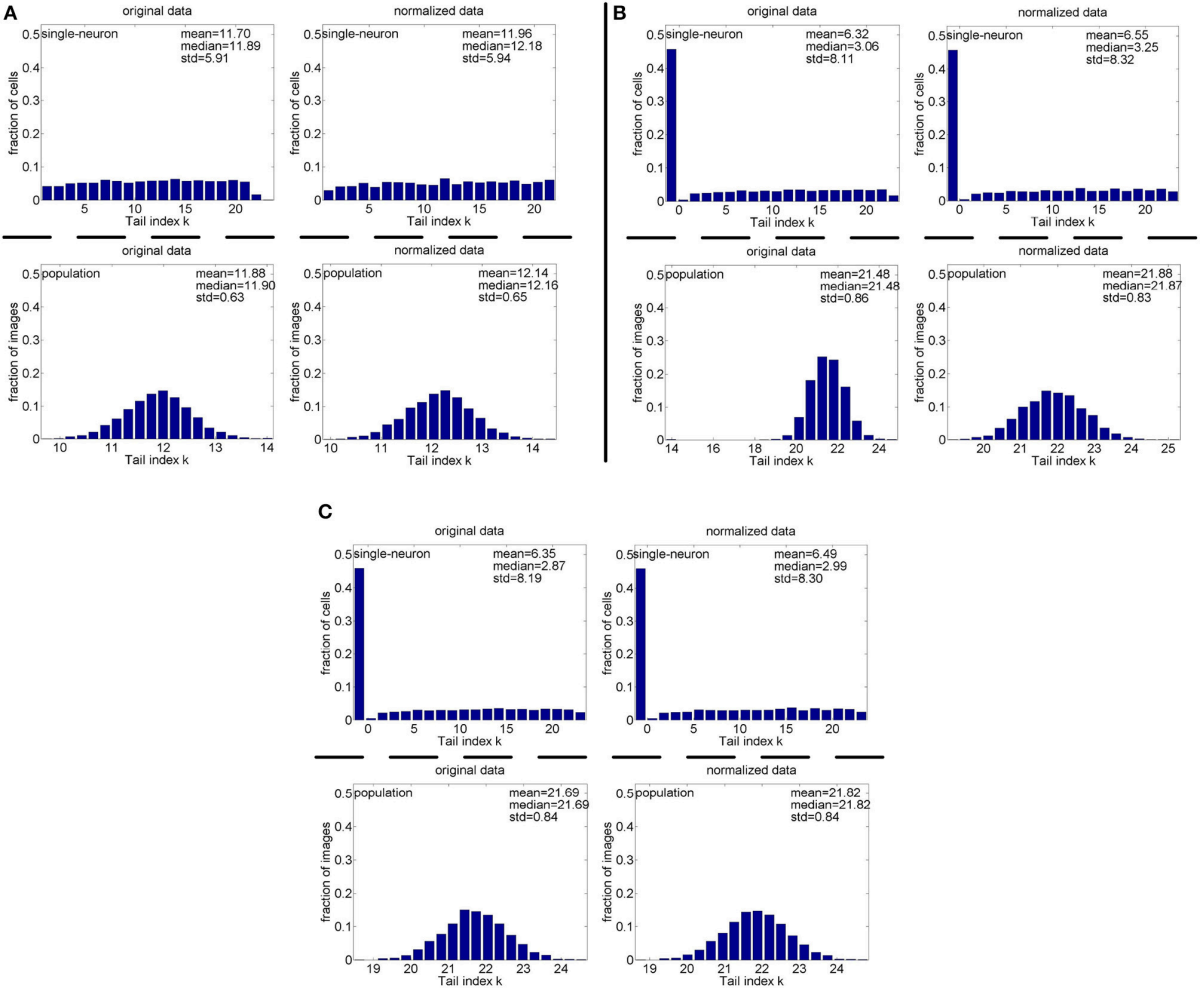
Histograms of the computed PTI-values of single-neuron responses and population responses in {*R*_1_, *R*_2_, *R*_3_} under Method-I: **(A)** Results on *R*_1_; **(B)** Results on *R*_2_; **(C)** Results on *R*_3_.

**Method-I:** Figure 10 shows the histograms of the computed PTI [i.e., *k* in Equation (3)] for the single-neuron responses and the population responses in the noiseless response matrices {*R*_1_, *R*_2_, *R*_3_}, and Columns 2–7 of Table 5 list the corresponding mean and median PTI on these responses. As is seen, the computed mean and median values of *k* for the normalized single-neuron responses is quite close to those for the unnormalized single-neuron responses. The standard deviations of *k* for the single-neuron responses are quite large, which are consistent with the corresponding kurtosis results in Section 3.2.1. The computed mean value of *k* for the unnormalized population responses is larger than that for the unnormalized single-neuron responses, which is inconsistent with the corresponding kurtosis results in Section 3.2.1. The computed median values of *k* for the unnormalized population responses is larger than that for the unnormalized single-neuron responses, and the computed mean and median values of *k* for the normalized population responses are also larger than those for the normalized single-neuron responses. These results are consistent with the corresponding kurtosis results in Section 3.2.1.

**Table 5:** Relative magnitude order of PTI between the single-neuron selectivity and the population sparseness on the unnormalized/normalized synthetic responses by Method-I.

	***R***_**1**_		***R***_**2**_		***R***_**3**_		${\tilde{R}}_{2}^{1}$		${\tilde{R}}_{2}^{2}$		${\tilde{R}}_{2}^{3}$	
	**Mean**	**Median**	**Mean**	**Median**	**Mean**	**Median**	**Mean**	**Median**	**Mean**	**Median**	**Mean**	**Median**
**UNNORMALIZED DATA**												
Single-neuron selectivity	11.70	11.89	6.32	3.06	6.35	2.87	0.26	−0.09	−0.02	−0.13	0.01	−0.09
Relative magnitude order	∧	∧	∧	∧	∧	∧	∧	∧	∧	∧	∧	∧
Population sparseness	11.88	11.90	21.48	21.48	21.69	21.69	1.76	1.76	0.75	0.75	0.76	0.76
**NORMALIZED DATA**												
Single-neuron selectivity	11.96	12.18	6.55	3.25	6.49	2.99	0.26	−0.09	−0.02	−0.13	0.01	−0.09
Relative magnitude order	∧	∨	∧	∧	∧	∧	∧	∧	∧	∧	∧	∧
Population sparseness	12.14	12.16	21.88	21.87	21.82	21.82	0.38	0.37	0.18	0.18	0.63	0.63

∧ *Indicates the selectivity is lower than the corresponding sparseness*.

**Figure 11:**
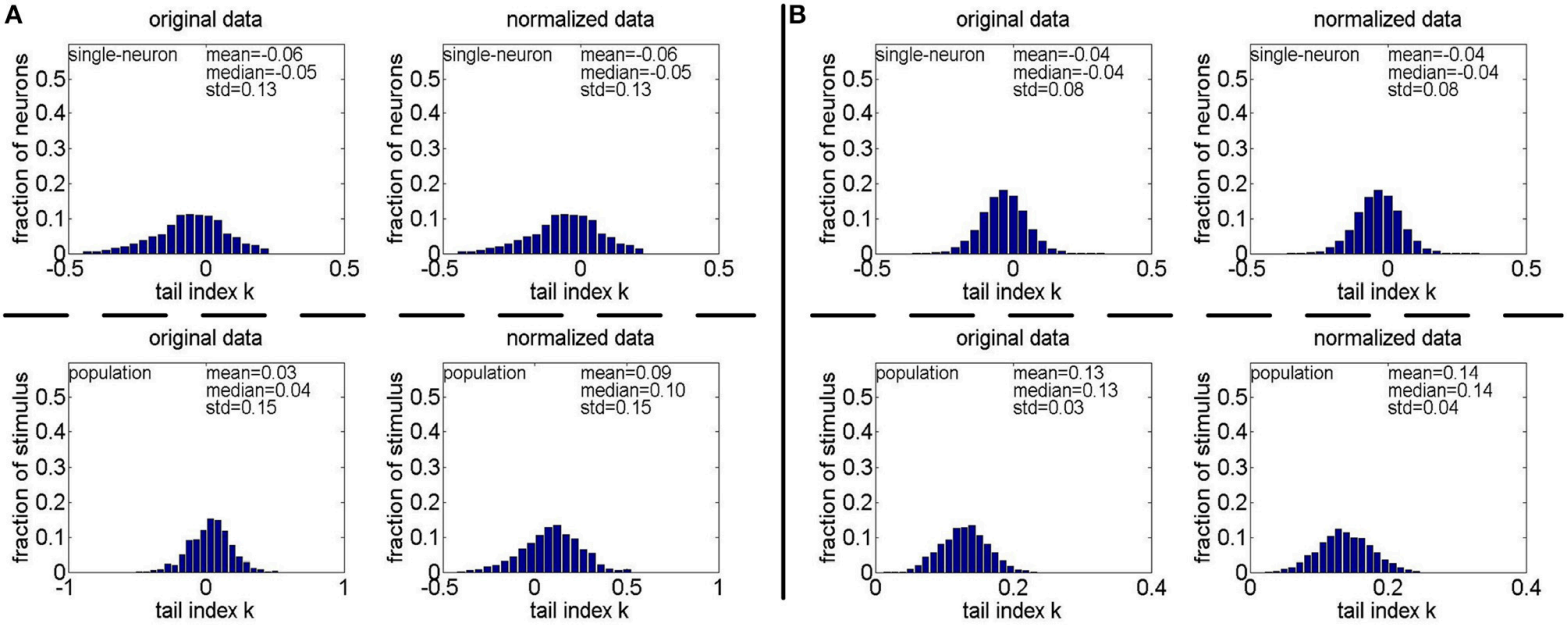
Histograms of the computed PTI-values of single-neuron responses and population responses in {*R*_4_, *R*_5_} under Method-II: **(A)** Results on *R*_4_; **(B)** Results on *R*_5_.

**Method-II:** Figure 11 shows the histograms of *k* for the single-neuron responses and the population responses in {*R*_4_, *R*_5_}, and Columns 2–5 of Table 6 list the corresponding mean and median PTI on these responses. As is seen, for both the unnormalized and normalized data, the computed mean and median values of *k* for the population sparseness are larger than those for the single-neuron selectivity. These results are consistent with the corresponding PTI results on {*R*_1_, *R*_2_, *R*_3_} reported in Table 5.

**Table 6:** Relative magnitude order of PTI between the single-neuron selectivity and the population sparseness on the unnormalized/normalized synthetic responses generated by Method-II.

	***R***_**4**_		***R***_**5**_		${\tilde{R}}_{4}^{2}$		${\tilde{R}}_{5}^{2}$	
	**Mean**	**Median**	**Mean**	**Median**	**Mean**	**Median**	**Mean**	**Median**
**UNNORMALIZED DATA**								
Single-neuron selectivity	−0.06	−0.05	−0.04	−0.04	−0.05	−0.04	−0.04	−0.04
Relative magnitude order	∧	∧	∧	∧	∧	∧	∧	∧
Population sparseness	0.03	0.04	0.13	0.13	0.10	0.10	0.10	0.10
**NORMALIZED DATA**								
Single-neuron selectivity	−0.06	−0.05	−0.04	−0.04	−0.05	−0.04	−0.04	−0.04
Relative magnitude order	∧	∧	∧	∧	∧	∧	∧	∧
Population sparseness	0.09	0.10	0.14	0.14	0.07	0.08	0.10	0.10

∧ *Indicates the selectivity is lower than the corresponding sparseness*.

#### 3.3.2. Pareo tail index for responses with noise

**Figure 12:**
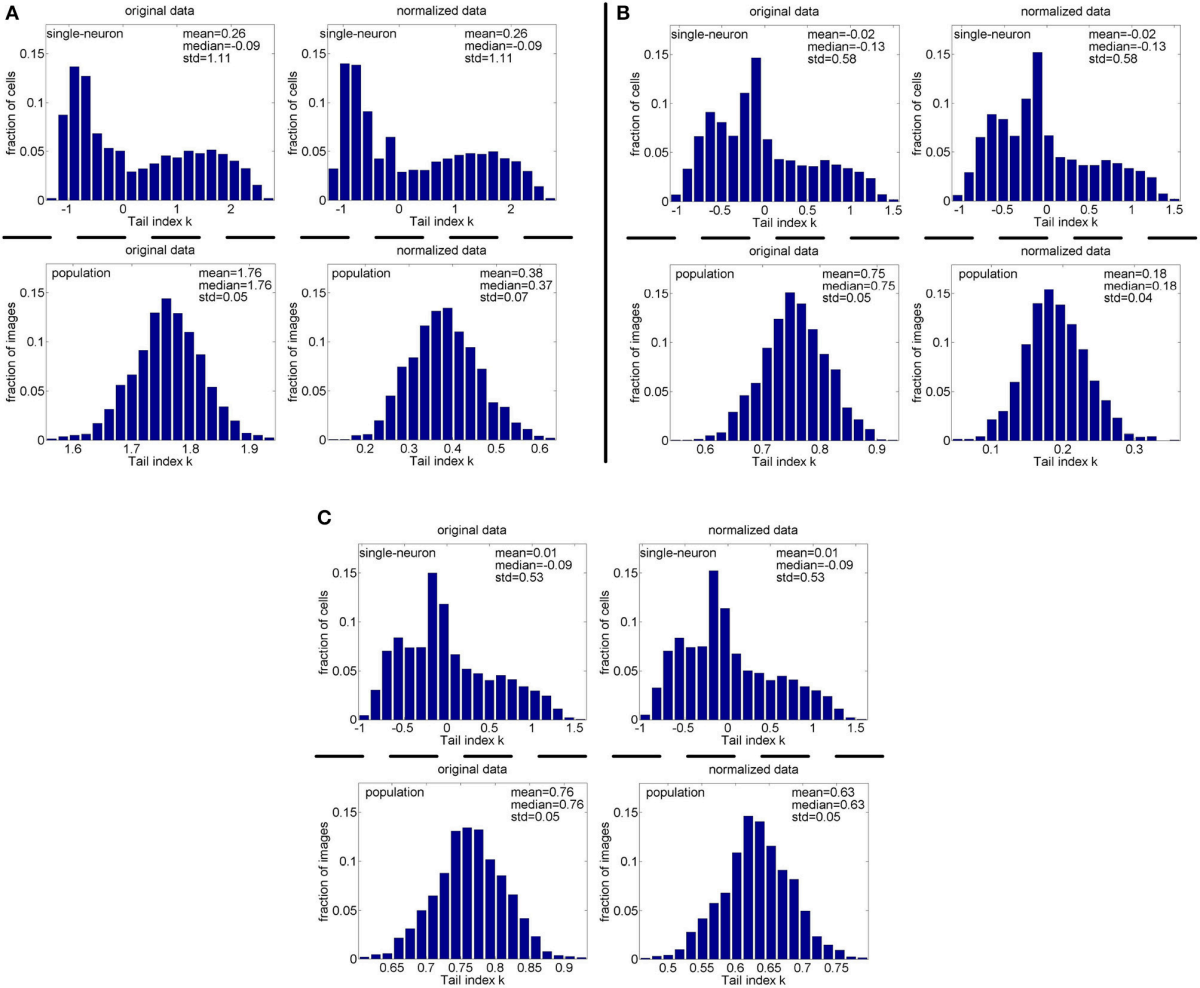
Histograms of the computed PTI-values of single-neuron responses and population responses in $\left\{{\tilde{R}}_{2}^{1},{\tilde{R}}_{2}^{2},{\tilde{R}}_{2}^{3}\right\}$ under Method-I: **(A)** Results on ${\tilde{R}}_{2}^{1}$; **(B)** Results on ${\tilde{R}}_{2}^{2}$; **(C)** Results on ${\tilde{R}}_{2}^{3}$.

**Method-I:** Figure 12 shows the histograms of the computed PTI for the single-neuron responses and the population responses in the noisy response matrices $\left\{{\tilde{R}}_{3}^{1},{\tilde{R}}_{3}^{2},{\tilde{R}}_{3}^{3}\right\}$, and Columns 8–13 of Table 5 list the corresponding mean and median PTI on these responses. As is seen, the computed mean and median values of *k* for the normalized single-neuron responses is close to those for the unnormalized single-neuron responses. And for both the unnormalized and normalized data, the computed mean and median values of *k* for the population sparseness are larger than those for the single-neuron selectivity. These results are all consistent with the corresponding kurtosis results in Section 3.2.2.

**Figure 13:**
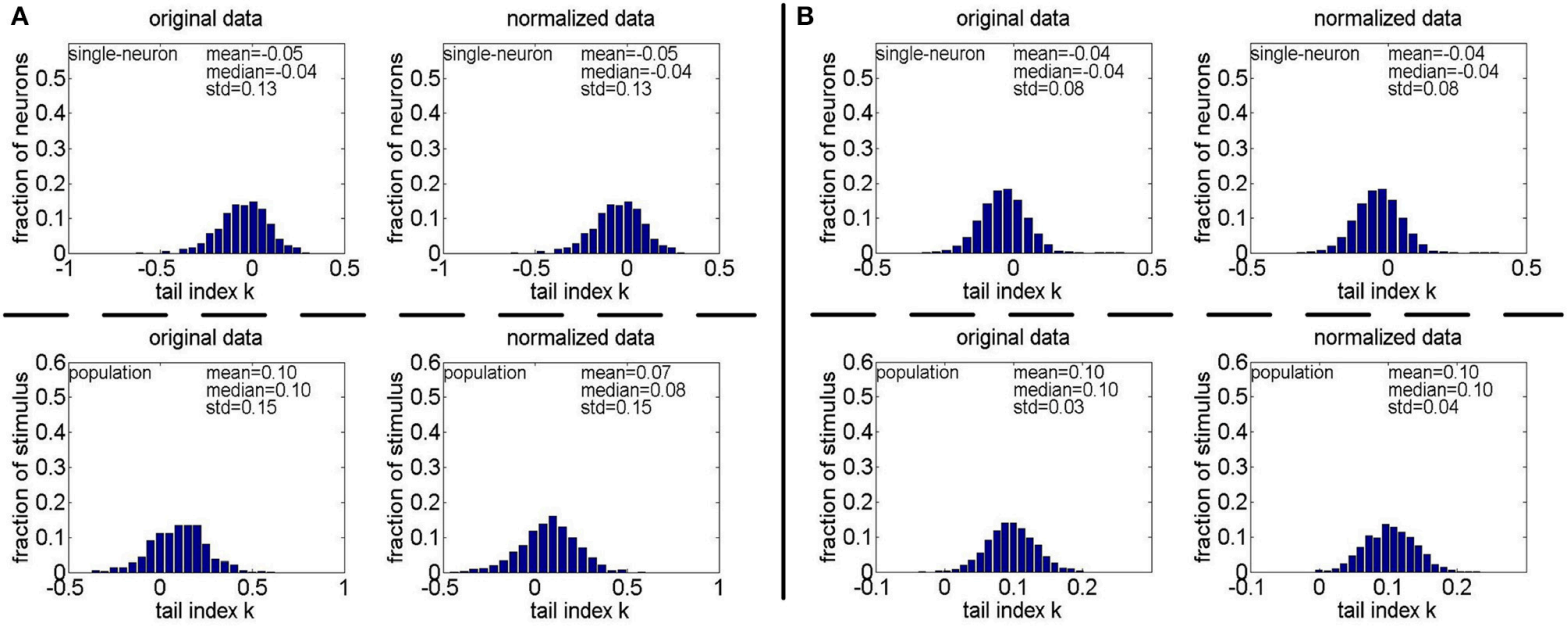
Histograms of the computed PTI-values of single-neuron responses and population responses in $\left\{{\tilde{R}}_{4}^{2},{\tilde{R}}_{5}^{2}\right\}$ under Method-II: **(A)** Results on ${\tilde{R}}_{4}^{2}$; **(B)** Results on ${\tilde{R}}_{5}^{2}$.

**Method-II:** As indicated in Section 2.3.2.2, the PTI is too sensitive to the Poisson-noise responses generated by Method-II, hence, we only report the results on the truncated-Gaussian-noise responses in $\left\{{\tilde{R}}_{4}^{2},{\tilde{R}}_{5}^{2}\right\}$ here (the results on the Poisson-noise responses in $\left\{{\tilde{R}}_{5}^{1},{\tilde{R}}_{5r1}^{1},{\tilde{R}}_{5r2}^{1}\right\}$ are reported in Figure S4 of the Supplementary Materials). Figure 13 shows the histograms of *k* for the single-neuron responses and the population responses in $\left\{{\tilde{R}}_{4}^{2},{\tilde{R}}_{5}^{2}\right\}$, and Columns 6–9 of Table 6 list the corresponding mean and median PTI on these responses. As is seen, once again, for both unnormalized and normalized responses, the computed mean and median values of *k* for the population sparseness are larger than those for the single-neuron selectivity. These results are all consistent with the results on $\left\{{\tilde{R}}_{3}^{1},{\tilde{R}}_{3}^{2},{\tilde{R}}_{3}^{3}\right\}$ reported in Table 5.

In sum, similar to the results with the kurtosis measure (except for the unnormalized noiseless data), the estimated mean and median PTI for the population sparseness is larger than that for the single-neuron selectivity, which is consistent with the statistics for AIT neurons in Lehky et al. (2011). Since noise is inevitable in real neuron recording, we thought our results on noisy data would be more informative.

#### 3.3.3. Pareto tail index for responses with neural correlation

The PTI-values of the unnormalized and normalized responses with neural correlations in $\left\{R_{5r1},R_{5r2},{\tilde{R}}_{5r1}^{2},{\tilde{R}}_{5r2}^{2}\right\}$ are computed here. The corresponding results are reported in Table 7 (also in Figure S5 of the Supplementary Materials). As is seen, for both unnormalized and normalized data, the computed mean and median values of *k* for the population sparseness are larger than those for the single-neuron selectivity. These results are consistent with the results on the responses without neural correlations reported in Table 6.

**Table 7:** Relative magnitude order of PTI between the single-neuron selectivity and the population sparseness on the unnormalized/normalized synthetic responses with neural correlation.

	***R***_**5***r***1**_		***R***_**5***r***2**_		${\tilde{R}}_{5r1}^{2}$		${\tilde{R}}_{5r2}^{2}$	
	**Mean**	**Median**	**Mean**	**Median**	**Mean**	**Median**	**Mean**	**Median**
**UNNORMALIZED DATA**								
Single-neuron selectivity	−0.04	−0.03	−0.04	−0.03	−0.04	−0.04	−0.03	−0.03
Relative magnitude order	∧	∧	∧	∧	∧	∧	∧	∧
Population sparseness	0.13	0.13	0.10	0.10	0.08	0.08	0.09	0.08
**NORMALIZED DATA**								
Single-neuron selectivity	−0.04	−0.03	−0.04	−0.03	−0.04	−0.04	−0.03	−0.03
Relative magnitude order	∧	∧	∧	∧	∧	∧	∧	∧
Population sparseness	0.12	0.12	0.12	0.12	0.10	0.10	0.09	0.09

∧ *Indicates the selectivity is lower than the corresponding sparseness*.

## 4. Discussion

The main objective of this work is to assess whether the population sparseness is always larger than the single-neuron selectivity by simulation under the condition that each neuron indeed only responds to a very limited number of stimuli among a very large number of stimuli. More specially, we would investigate:

1. Whether the mean (median) kurtosis for single-neuron selectivity is always smaller than the mean (median) kurtosis for population sparseness for both the normalized data and unnormalized data as did the AIT neuron responses in monkey in Lehky et al. (2011);
2. Whether the mean (median) PTI for single-neuron selectivity is always smaller than the mean (median) PTI for population sparseness for both the normalized data and unnormalized data as did the AIT neuron responses in monkey in Lehky et al. (2011).

To address the above two issues, two different neuron response generating methods are explored. Method-I can explicitly control the small number of selective stimuli of individual neurons, but lacks any biological basis. Method-II is an approximation of monkey IT neuron response (Lehky et al., 2011), but can only constrain implicitly the sparseness of selective stimuli of individual neurons by the gamma distribution. Arguably different generating approaches could affect final statistics, however, the obtained results under both Method-I and Method-II are largely consistent in most cases. Our results show that: even with the stimulus number and neuron number in the order of several thousands rather than several hundreds as reported in Lehky et al. (2011), the relative magnitude order of mean kurtosis (median kurtosis, mean PTI, and median PTI) for the single-neuron selectivity with respect to the mean kurtosis (median kurtosis, mean PTI, and median PTI) for the population sparseness is largely preserved for both the normalized and unnormalized data in most cases. This supports the interpretation of the AIT neuron response statistics in Lehky et al. (2011), and also implies that the results on 674 AIT neurons under 806 image stimuli in Lehky et al. (2011) capture the essential features of more AIT neurons in monkey for image object representation. In other words, if more AIT neurons were recorded, the above relative magnitude order would expect to keep unchanged.

Here, we would point out that by “consistency” or “agreement” in this text, we only mean the relative magnitude order of the four entities (mean kurtosis, median kurtosis, mean PTI, media PTI) for the single-neuron selectivity to the corresponding ones for the population sparseness keeps unchanged, we do not mean their absolute values of these entities are similar between the synthetic data and the AIT neuron data.

**Figure 14:**
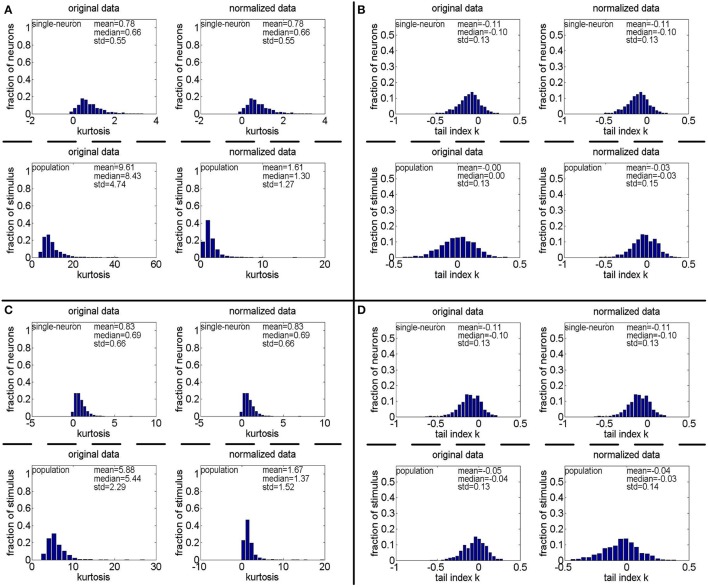
Kurtosis and PTI of responses generated by gamma distributions different from that used in Lehky et al. (2011). **(A,B)** Kurtosis and PTI by a gamma distribution with *a* = gamrnd(8.0, 1.0) and *b* = gamrnd(2.0, 0.5), respectively. **(C,D)** Kurtosis and PTI by a gamma distribution with *a* = gamrnd(8.0, 1.0) and *b* = gamrnd(3.0, 1.0), respectively.

We also found that how to generate a gamma-distribution parameter pair (*a, b*), in particular, the shape parameter *a*, is a crucial issue for Method-II. For this aspect, we computed the kurtosis and PTI on two extra 806 × 674 response matrices generated by a gamma distribution with {*a* = gamrnd(8.0, 1.0), *b* = gamrnd(2.0, 0.5)} and a gamma distribution with {*a* = gamrnd(8.0, 1.0), *b* = gamrnd(3.0, 1.0)}. The corresponding results are shown in Figure 14. Comparing Figures 4A, 11A (showing the kurtosis and PTI on *R*_4_ generated by a gamma distribution with {*a* = gamrnd(4.0, 0.5), *b* = gamrnd(2.0, 0.5)} as specified in Lehky et al. (2011)) with Figures 14A,B, we can see that for different choices of the shape parameter *a*, the obtained kurtosis and PTI are quite different. This means that merely knowing the gamma distribution of individual neuron response is not sufficient to evaluate the statistics of neuron population responses, currently it seems we still lack the theoretical basis of generating the suitable shape and scale parameter pairs of population neurons.

It is also found that for either kurtosis or PTI, the associated standard deviation for the single-neuron selectivity is quite large in some cases. This reveals some limitations of using them as neuron selectivity measures. From the mathematical point view, if the associated standard deviation is large, the mean, or median becomes less informative.

Clearly, noise also plays a role on the final estimations. In this work, different kinds of noise and different levels of noise are investigated, the results are rather consistent. Evidently, if the noise level is further increased, it will certainly and more severely affect the response statistics. As shown in Figure 6, by increasing the noise level, the kurtosis statistics decrease accordingly. This suggests that for AIT neuron response statistics, how to appropriately estimate the noise level should be carefully considered taking into the account of different recorded neuron sites, recording timing and subjects.

Neuron response correlation is another important issue of affecting the absolute value of kurtosis and PTI. However, our results show that neuron response correlation does not affect the relative magnitude order between the population sparseness and the single-neuron selectivity under the kurtosis and Pareto tail index criteria. Note also that, only linear correlation is simulated here, however, nonlinear high-order correlation surely must also affect the response statistics, which will be a more difficult issue, and is beyond our current work.

Finally, considering all the above listed factors as well as other possible factors, our current simulation work is merely a qualitative comparison of those reported neuron response statistics in Lehky et al. (2011). It cannot be excluded that our work is another story of “A blind man conceptualizing elephant by only touching and feeling the trunk.” With the advance of new neuron recording technology, more neurons could be recorded, and the mystery of neuron object representations will be further clarified in the future.

## Author contributions

ZH conceived of the comparison of IT neuron response statistics by simulation. QD and ZH explored the method. QD and BL implemented the explored method and performed the validation. QD and ZH wrote the paper.

### Conflict of interest statement

The authors declare that the research was conducted in the absence of any commercial or financial relationships that could be construed as a potential conflict of interest.
