# Supplementary material for: Comparison of IT Neural Response Statistics with Simulations
Source: Front Comput Neurosci. 2017 Jul 12;11:60. doi: 10.3389/fncom.2017.00060 (PMC5506183; doi:10.3389/fncom.2017.00060)
Supplement: Supplementary file 1 [file DataSheet1.pdf]

Supplementary Material: Comparison of IT Neural Response Statistics with Simulations

Qiulei Dong, Bo Liu, Zhanyi Hu *
*Correspondence:
Zhanyi Hu
huzy@nlpr.ia.ac.cn

1 SUPPLEMENTARY FIGURES

1.1 Figures

Figure S1 shows the computed mean, median, standard deviation(std) of the computed kurtosis values for the single-neuron responses and the population responses in the response matrices $\{R_{5r1}, R_{5r2}, \tilde{R}^1_{5r1}, \tilde{R}^1_{5r2}, \tilde{R}^2_{5r1}, \tilde{R}^2_{5r2}\}$ with correlation $r = \{0.1, 0.2\}$ under Method-II. As is seen, for both the unnormalized and normalized responses, the computed mean and median kurtosis values for the population sparseness are larger than those for the single-neuron selectivity.

Figure S2 shows the computed mean and median kurtosis for the single-neuron responses and the population responses with different numbers of stimuli and neurons on $\{R_5, R_{5r1}, R_{5r2}\}$ under Method-II, and Figure S3 shows the computed mean and median kurtosis for the single-neuron responses and the population responses with different numbers of stimuli and neurons on the Poisson-noise responses matrices $\{\tilde{R}^1_5, \tilde{R}^1_{5r1}, \tilde{R}^1_{5r2}\}$ under Method-II. As is seen, for both the unnormalized and normalized responses, the computed mean and median kurtosis values for the population sparseness are still larger than those for the single-neuron selectivity.

Figure S4 shows the histograms of the computed Pareto tail indices of the single-neuron responses and the population responses in the Poisson-noise response matrices $\{\tilde{R}^1_5, \tilde{R}^1_{5r1}, \tilde{R}^1_{5r2}\}$ under Method-II. As is seen, the mean value of the computed Pareto tail indices for the single-neuron responses is much larger the median value of the computed Pareto tail indices, and the standard deviations of the computed Pareto tail indices for the single-neuron responses are also quite large. This means that the Pareto tail index criterion is quite sensitive to the Poisson-noise responses.

Figure S5 shows the histograms of the computed Pareto tail indices of the single-neuron responses and the population responses in $\{R_{5r1}, R_{5r2}, \tilde{R}^2_{5r1}, \tilde{R}^2_{5r2}\}$ with correlation $r = \{0.1, 0.2\}$ under Method-II. As is seen, for both the unnormalized and normalized responses, the mean and median values of the computed Pareto tail indices for the population sparseness are always larger than those for the single-neuron selectivity.

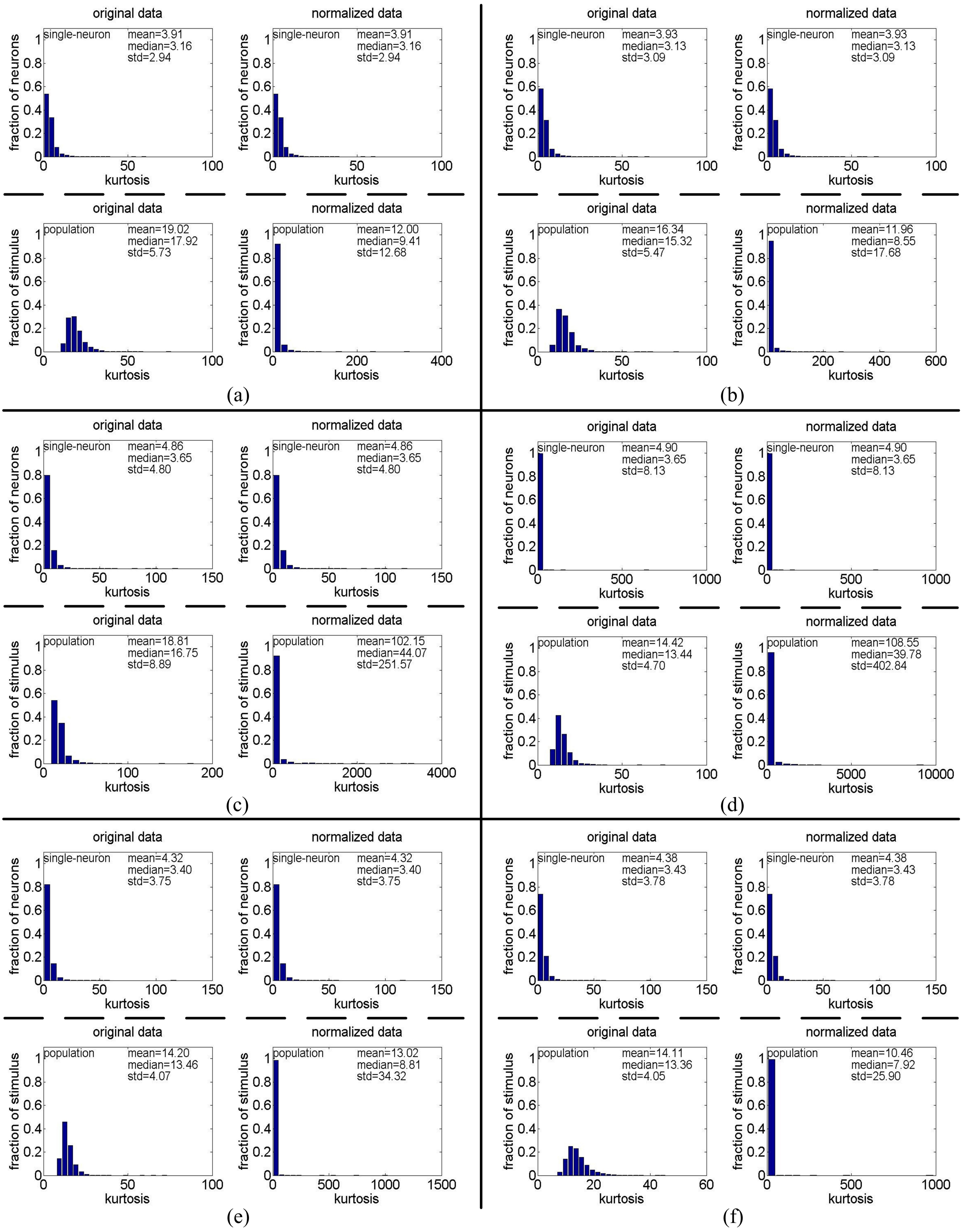

Figure S1. Single-neuron selectivity and population sparseness on the response matrices $\{R_{5r1}, R_{5r2}, \tilde{R}^1_{5r1}, \tilde{R}^1_{5r2}, \tilde{R}^2_{5r1}, \tilde{R}^2_{5r2}\}$ with neural correlation under Method-II: (a) Results on R_{5r1}; (b) Results on R_{5r2}; (c) Results on $\tilde{R}^1_{5r1}$; (d) Results on $\tilde{R}^1_{5r2}$; (e) Results on $\tilde{R}^2_{5r1}$; (f) Results on $\tilde{R}^2_{5r2}$.

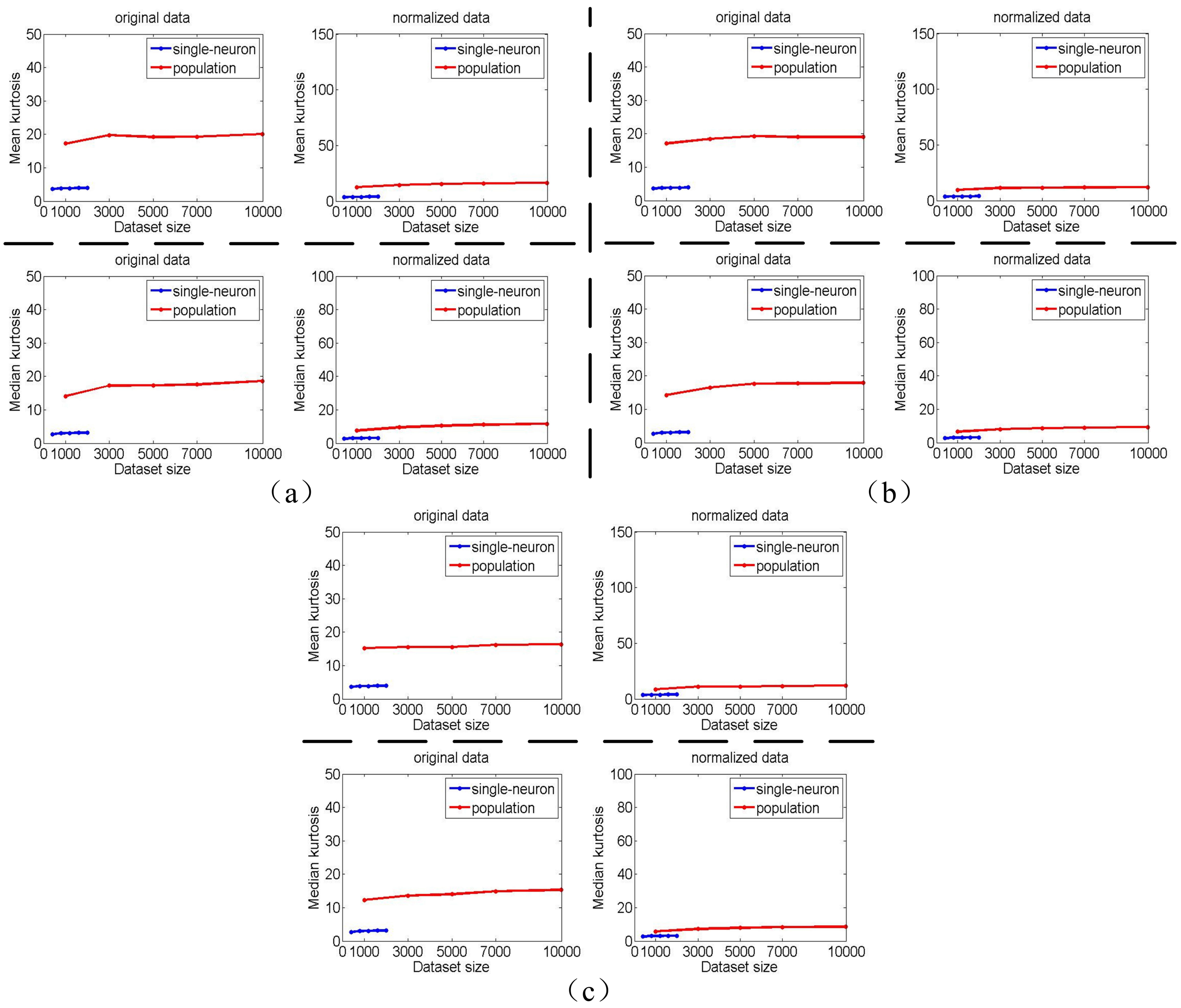

Figure S2. Mean and median kurtosis for single-neuron responses and population responses with different stimuli and neurons on $\{R_5, R_{5r1}, R_{5r2}\}$ under Method-II: (a) Results on R_5; (b) Results on R_{5r1}; (c) Results on R_{5r2}.

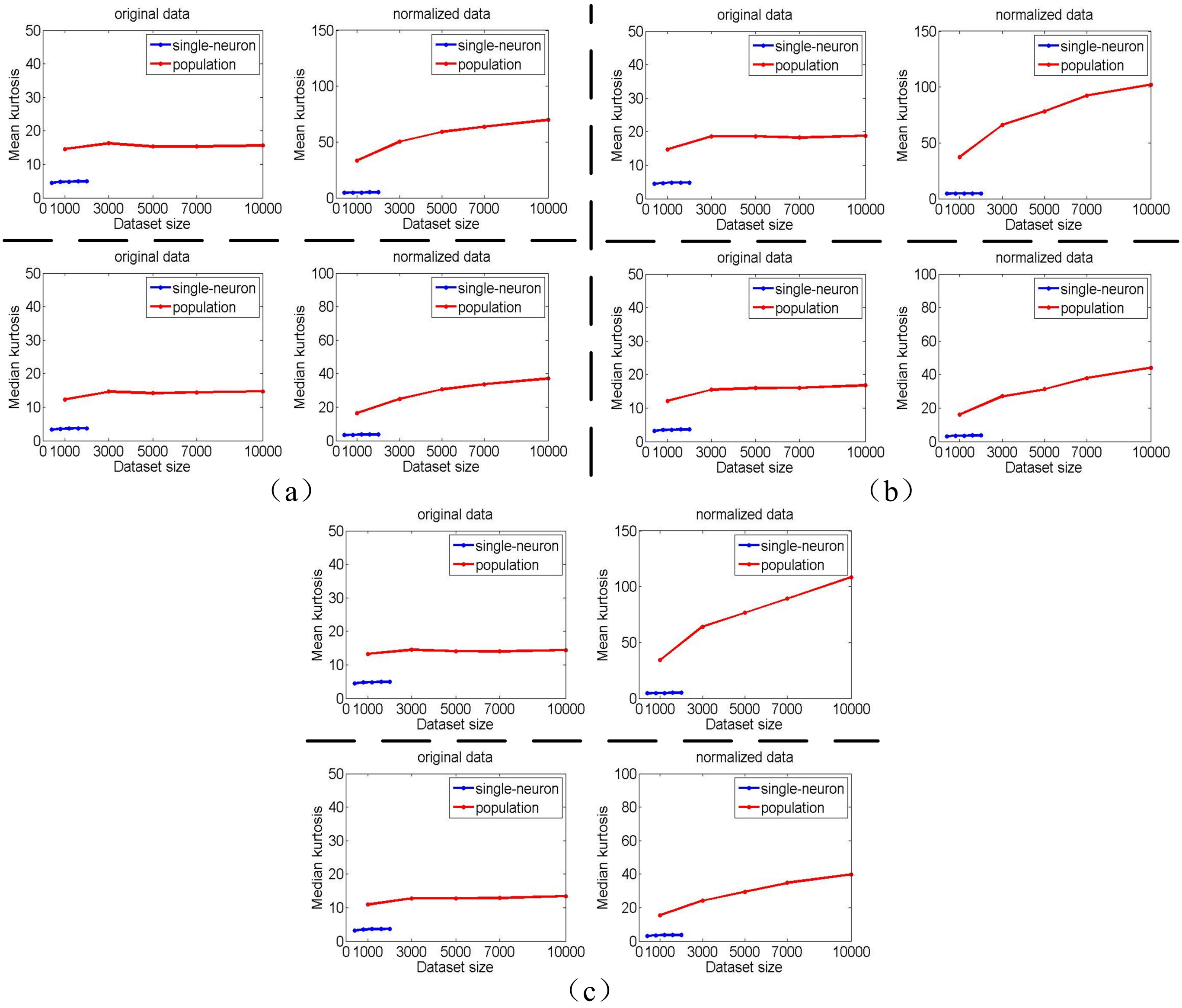

Figure S3. Mean and median kurtosis for single-neuron responses and population responses with different stimuli and neurons on $\{\tilde{R}^1_5, \tilde{R}^1_{5r1}, \tilde{R}^1_{5r2}\}$ under Method-II: (a) Results on $\tilde{R}^1_5$; (b) Results on $\tilde{R}^1_{5r1}$; (c) Results on $\tilde{R}^1_{5r2}$.

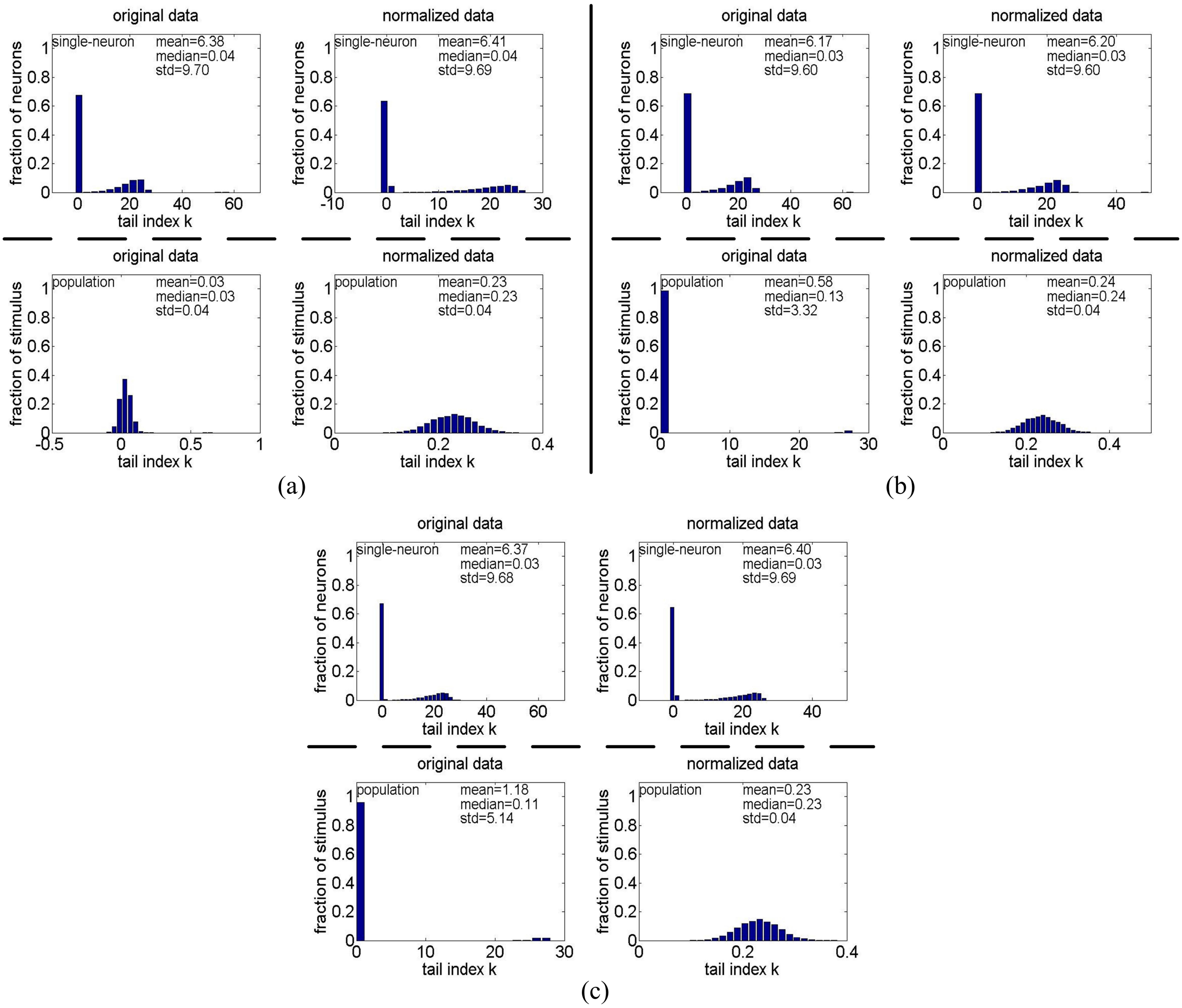

Figure S4. Histograms of the computed Pareto tail indices of single-neuron responses and population responses in the Poisson-noise response matrices $\{\tilde{R}_5^1, \tilde{R}_{5r1}^1, \tilde{R}_{5r2}^1\}$ under Method-II: (a) Results on $\tilde{R}_5^1$; (b) Results on $\tilde{R}_{5r1}^1$; (c) Results on $\tilde{R}_{5r2}^1$.

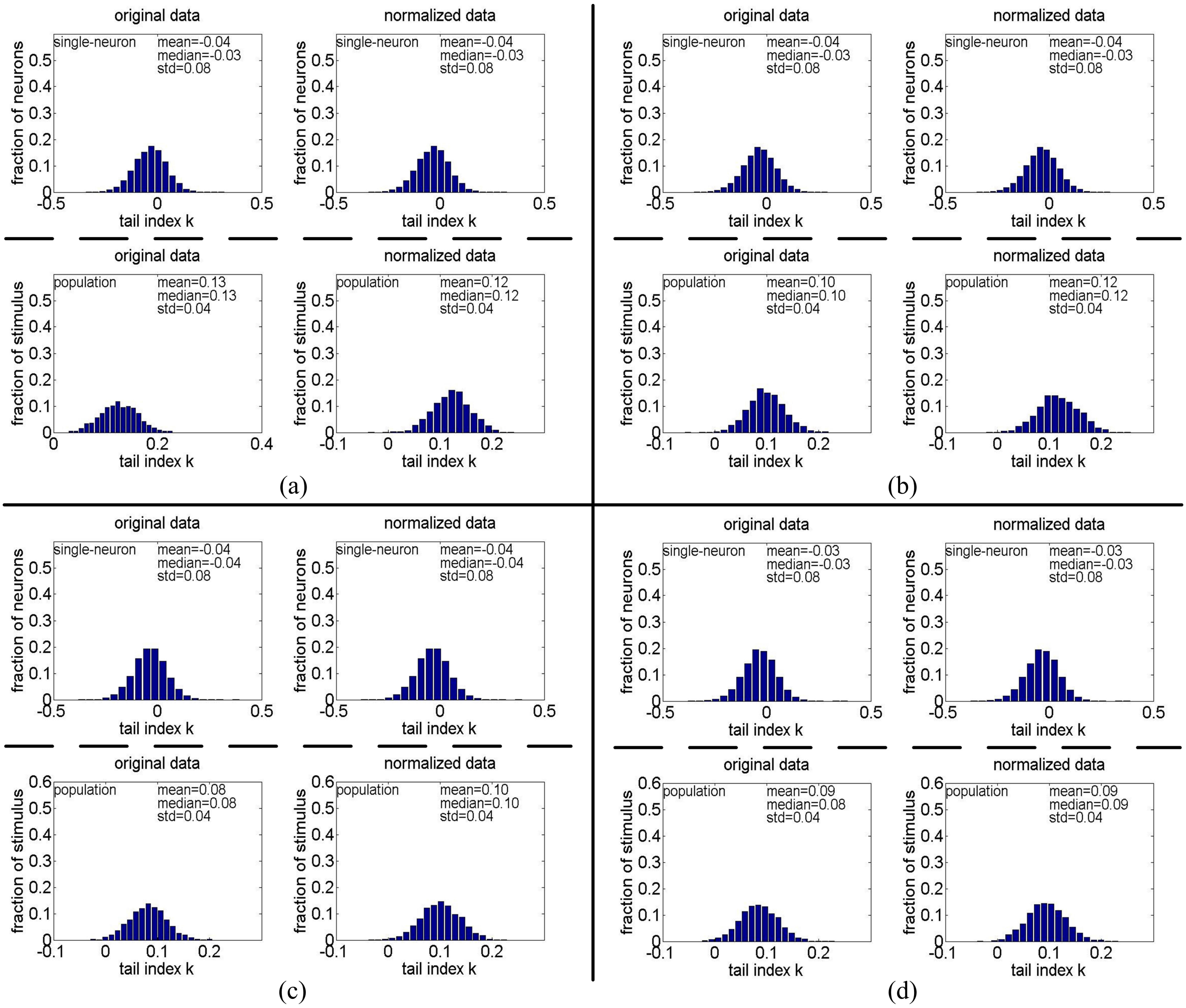

Figure S5. Histograms of the computed Pareto tail indices of single-neuron responses and population responses in $\{R_{5r1}, R_{5r2}, \tilde{R}^2_{5r1}, \tilde{R}^2_{5r2}\}$ under Method-II: (a) Results on R_{5r1}; (b) Results on R_{5r2}; (c) Results on $\tilde{R}^2_{5r1}$; (d) Results on $\tilde{R}^2_{5r2}$.
